# Highly efficient AlGaN-based deep-ultraviolet light-emitting diodes: from bandgap engineering to device craft

**DOI:** 10.1038/s41378-024-00737-x

**Published:** 2024-08-13

**Authors:** Xu Liu, Zhenxing Lv, Zhefu Liao, Yuechang Sun, Ziqi Zhang, Ke Sun, Qianxi Zhou, Bin Tang, Hansong Geng, Shengli Qi, Shengjun Zhou

**Affiliations:** 1 Center for Photonics and Semiconductors, School of Power and Mechanical Engineering, Wuhan University, Wuhan, 430072 China; 2 Ningbo ANN Semiconductor Co. Ltd., Ningbo, 315336 China; 3 The Institute of Technological Sciences, Wuhan University, Wuhan, 430072 China

**Keywords:** Optical materials and structures, Electrical and electronic engineering

## Abstract

AlGaN-based light-emitting diodes (LEDs) operating in the deep-ultraviolet (DUV) spectral range (210–280 nm) have demonstrated potential applications in physical sterilization. However, the poor external quantum efficiency (EQE) hinders further advances in the emission performance of AlGaN-based DUV LEDs. Here, we demonstrate the performance of 270-nm AlGaN-based DUV LEDs beyond the state-of-the-art by exploiting the innovative combination of bandgap engineering and device craft. By adopting tailored multiple quantum wells (MQWs), a reflective Al reflector, a low-optical-loss tunneling junction (TJ) and a dielectric SiO_2_ insertion structure (IS-SiO_2_), outstanding light output powers (LOPs) of 140.1 mW are achieved in our DUV LEDs at 850 mA. The EQEs of our DUV LEDs are 4.5 times greater than those of their conventional counterparts. This comprehensive approach overcomes the major difficulties commonly faced in the pursuit of high-performance AlGaN-based DUV LEDs, such as strong quantum-confined Stark effect (QCSE), severe optical absorption in the p-electrode/ohmic contact layer and poor transverse magnetic (TM)-polarized light extraction. Furthermore, the on-wafer electroluminescence characterization validated the scalability of our DUV LEDs to larger production scales. Our work is promising for the development of highly efficient AlGaN-based DUV LEDs.

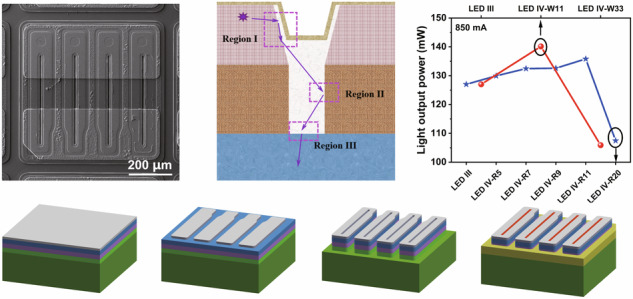

## Introduction

Deep-ultraviolet (DUV) germicidal irradiation is a chemical-free, species-agnostic disinfection treatment because DUV light with a wavelength between 200 and 280 nm can achieve sterilization by damaging the deoxyribonucleic/ribonucleic acid of pathogens^1–3^. Compared with conventional mercury lamps, AlGaN-based DUV light-emitting diodes (LEDs) have a longer lifetime, greater stability, more environmentally friendly manufacturing and more flexible operation; thus, these LEDs have attracted much academic and commercial interest as the most competitive candidates for physical disinfection in recent years^4–6^. However, many technical challenges hinder the further development of AlGaN-based DUV LEDs and include poor external quantum efficiency (EQE), poor light extraction efficiency (LEE), strong quantum-confined Stark effect (QCSE) and large DUV light absorption due to the p-GaN ohmic contact layer and opaque p-electrode^7^. Among these issues, the course of achieving high-performance DUV LEDs is accompanied by strong QCSE and poor LEE.

In Al_x_Ga_1-x_N/Al_y_Ga_1-y_N multiple quantum wells (MQWs), lattice and thermal mismatches occur between the Al_x_Ga_1-x_N quantum well (QW) and Al_y_Ga_1-y_N quantum barrier (QB). Therefore, the Al_x_Ga_1-x_N QWs suffer from in-plane compressive stress, leading to a large piezoelectric polarization field between the QW and QB and tilting of the energy band of the MQW. This phenomenon is called QCSE^8^. The QCSE can suppress carrier radiative recombination by separating holes and electrons and reducing carrier wavefunction overlap, thereby decreasing the internal quantum efficiency (IQE). In addition, the topmost valence sub-band near the Γ point is the crystal-field splitting band because of the spin-orbital and crystal-field splitting effects^9–11^, thus leading to laterally propagating transverse magnetic (TM)-polarized light dominating the high-Al-content Al_x_Ga_1-x_N/Al_y_Ga_1-y_N MQWs. To date, numerous studies have been performed to resolve these problems; these studies include employing SiO_2_-antireflection films^12^, modifying electrodes^13–15^, regulating polarization electric fields^16–18^, utilizing plasmonic structures or photonic crystals^19–21^, using high-quality templates^22–26^, and applying micro-/nano-LED geometries^27–30^. Nevertheless, a comprehensive solution to inhibit the QCSE and simultaneously increase the LEE of DUV LEDs is still lacking.

In this study, we merged bandgap engineering with device craft to systematically improve the emission performance of an AlGaN-based DUV LED at an emission wavelength of ~270 nm. First, a tailored QW structure is proposed to neutralize the QCSE and improve the electron-hole wavefunction overlap, thereby enhancing the IQE of the DUV LED. Then, the low-optical-loss structure, composed of an AlGaN tunneling junction (TJ) and a reflective p-electrode, is introduced into the DUV LED to suppress DUV light absorption. Furthermore, the SiO_2_ insertion structure (IS-SiO_2_), which not only increases the effective current injected into the active region but also manipulates light transmission to enhance the emission of the top facet, is embedded into the DUV LED to enhance the LEE. Our study establishes a universal strategy for efficient DUV LEDs on sapphire, with the aim to develop high-quality lighting sources.

## Epitaxial growth of DUV LED structure

**Fig. 1:**
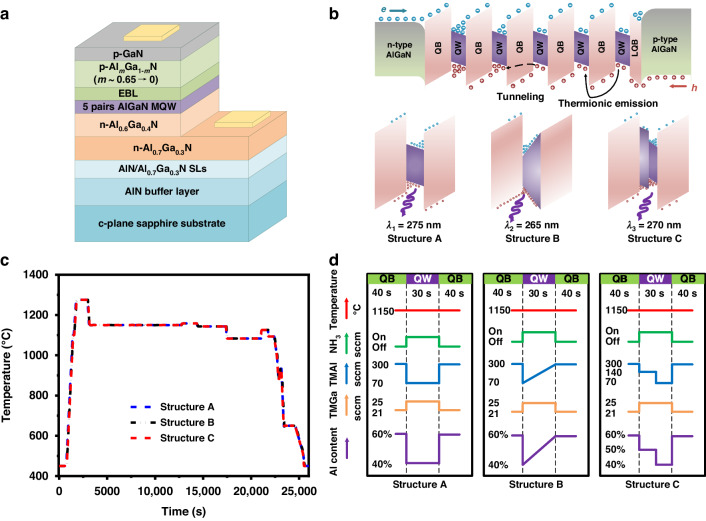
Epitaxial growth of the DUV LEDs with tailored QWs. Schematic illustration of the (**a**) AlGaN-based DUV LED and (**b**) corresponding energy band diagram of the tailored MQWs. **c** In situ temperature transients during the epitaxial growth of Structures A, B, and C. **d** Schematic illustration of the growth conditions of QW in Structures A, B, and C

The DUV LEDs were all grown on a *c*-plane sapphire substrate by using a metal-organic chemical vapor deposition system (Promaxy UV). The group-III element sources used in the experiment included trimethylgallium (TMGa) and trimethylaluminum (TMAl). High-purity ammonia (NH_3_) was used as the group-V element source. The dopants utilized to grow n-type and p-type layers are silane (SiH_4_) and bis(cyclopentadienyl)magnesium (Cp_2_Mg), respectively. Hydrogen (H_2_) served as a carrier gas to grow AlGaN, while H_2_ and nitrogen (N_2_) served as carrier gases to grow p-GaN. Figure 1a shows a schematic illustration of the AlGaN-based DUV LED. The epitaxial films of the DUV LED include the following layers from the bottom up: a 3-μm-thick undoped AlN buffer layer, a 410-nm-thick AlN/Al_0.7_Ga_0.3_N superlattice insertion layer, a 650-nm-thick n-type Al_0.7_Ga_0.3_N transition layer, an ~1-μm-thick n-type Al_0.6_Ga_0.4_N electron source layer, five pairs of Al_x_Ga_1-x_N/Al_0.68_Ga_0.32_N MQW, a 2-nm-thick Al_0.68_Ga_0.32_N last quantum barrier, a 40-nm-thick p-type Al_0.65_Ga_0.35_N electron blocking layer (EBL), a 44-nm-thick p-type Al_m_Ga_1-m_N (*m* ~ 0.65 → 0) hole source layer and an ~40-nm-thick p-type GaN ohmic contact layer. DUV LEDs with conventional, gradient and staggered QWs are labeled as Structures A, B and C, respectively, as shown in Fig. 1b. Compared to the conventional QWs in Structure A, the tailored QWs in Structures B and C are anticipated to enhance the radiative recombination rate of electron-hole pairs by alleviating the QCSE, thereby improving the emission performance of the device. The in situ temperature transients are shown in Fig. 1c. The growth temperatures of the MQWs in Structures A, B and C are all maintained at 1150 °C, which confirms that the growth temperature has a negligible impact on the change in the QW structure. Figure 1d illustrates the growth conditions of the QW in Structures A, B and C. We trim the QW structures only by adjusting the flux of TMAl.

The cross-sectional scanning electron microscopy (SEM) image of the epitaxial wafer and the transmission electron microscopy (TEM) image of the related MQW are shown in Fig. S1a, b (Supporting Information). The thicknesses of the QW and QB are 2.3 and 3.7 nm, respectively. Using an as-grown DUV wafer, we fabricated DUV LEDs via standard LED fabrication processes. The optical microscope image of a lighted DUV LED is shown in Fig. S1c (Supporting Information). The emission performance of the packaged DUV LED on the printed circuit board is shown in Fig. S1d (Supporting Information).

## Results and discussion

### Tailoring of the MQW structure and its application in DUV LEDs

High-resolution X-ray diffraction rocking curve measurements were performed to characterize the crystalline quality of the DUV wafers, as shown in Fig. S2a, b (Supporting Information). The full width at half-maximums (FWHMs) of the (002) and (102) reflection peaks are related to the dislocation density^31^. The FWHMs of the (002)/(102) reflection peaks for Structures A, B and C are 260/460, 260/460 and 280/480 arcsec, respectively. The functional relationship between the dislocation density and FWHM can be plotted by using Equation S1^32^ in Supplementary Note I. The estimated dislocation densities for Structures A, B and C are 4.36 × 10^9^, 4.36 × 10^9^ and 5.19 × 10^9 ^cm^−2^, respectively. Clearly, tailoring the QW structure by varying the Al content only exerts an inconsequential influence on the crystalline quality of the epitaxial wafer.

The strain states of Structures A, B and C were studied by room-temperature Raman scattering, and the results are shown in Fig. S3 (Supporting Information). For Structure A, the phonon modes near 599.8 cm^−1^ and 658.7 cm^−1^ correspond to the E^H^_2_ (GaN-like) mode and the E^H^_2_ (AlN-like) mode, respectively; this result confirms the presence of Al_0.6_Ga_0.4_N^33,34^. Compared to Structure A, the Raman peaks of the E^H^_2_ (AlN-like) mode for Structures B and C are at 659.7 and 658.7 cm^−1^, respectively. The Raman peak in Structure B shifts to the right and indicates an increasing compressive stress in comparison to those in Structures A and C. In contrast to Structure A, almost no variation in the stress state is observed in Structure C. The in-plane compressive stress can be calculated by using Equation S2 in Supplementary Note I. Compared to that of Structure A, the frequency of Structure B shifts to right and indicates the existence of an additional compressive stress of 270 MPa in the epitaxial layers.

**Fig. 2:**
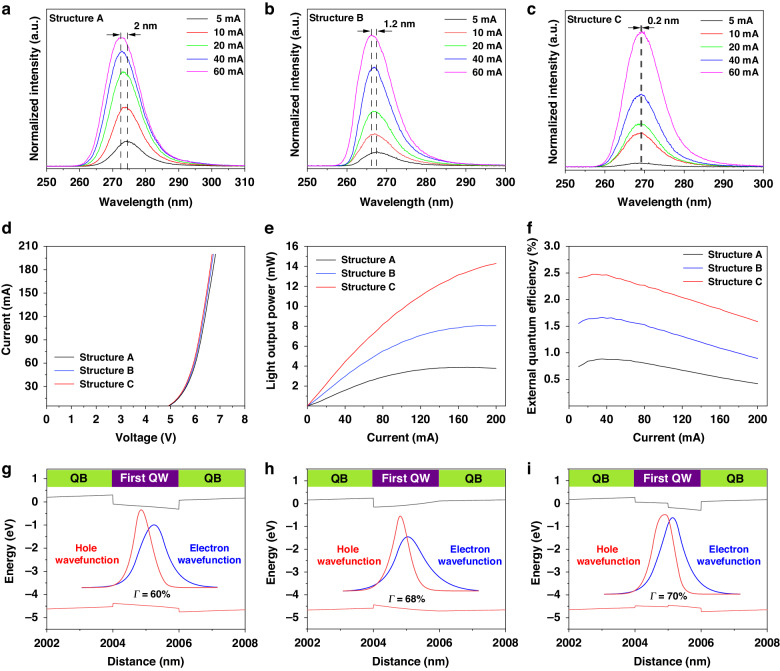
Electroluminescence properties of the DUV LEDs with tailored QWs. Normalized EL intensity of (**a**) Structure A, (**b**) Structure B, and (**c**) Structure C at various injection currents. **d** *I-V*, **e** LOP-I, and **f** EQE-I characteristics at injection currents from 0 to 200 mA. Calculated wavefunction overlap in the first QW region of (**g**) Structure A, (**h**) Structure B, and (**i**) Structure C at 5 V

To investigate the optical and electrical properties of DUV LEDs with different QW structures, electroluminescence (EL) and light output power (LOP)–current–voltage (L-I-V) characteristics for DUV LED with a size of 10 × 20 mil^2^ were measured via an integrating sphere using a semiconductor parameter analyzer (Keithley 4200) at room temperature. The EL spectra of Structures A, B and C at various injection currents are shown in Fig. 2a–c. At an injection current of 60 mA, the blue shifts of the peak wavelengths for Structures A, B and C are 2, 1.2 and 0.2 nm, respectively. Thus, the QCSE in the MQWs of Structures B and C are effectively alleviated in comparison to those in the MQWs of Structure A. In addition, barely any blue shift in the peak wavelength for Structure C is observed; this result indicates that the QCSE is almost completely suppressed in the MQWs of Structure C. At 60 mA, the FWHMs of Structures A, B and C are 12.1, 10.2, and 10.2 nm, respectively. The narrower FWHMs of Structures B and C are attributed to an alleviated QCSE, further verifying the advantage of the tailored MQW structure.

LOP-current-voltage (L-I-V) characterization analysis was performed to further investigate the luminescence properties of Structures A, B and C. The locations of all chips on Structures A, B, and C and the corresponding LOPs are plotted in Fig. S4 (Supporting Information). The chips are selected from the corresponding region in red and packaged to measure the L-I-V characteristics. Figure 2d shows the I‒V curves of packaged Structures A, B and C. The forward voltages of Structures A, B and C are 5.71, 5.73 and 5.79 V at 40 mA, respectively. Apparently, the modifications of the MQWs in Structures B and C do not damage the electrical properties of the device. The LOP is plotted as a function of the injection current for Structures A, B and C in Fig. 2e. At 40 mA, the LOPs of Structures A, B and C are 1.6, 3.1 and 3.9 mW, respectively. Notably, the LOPs of Structure B and Structure C are 2.4- and 3.9-fold greater than that of Structure A at 200 mA, respectively. Figure 2f shows the EQEs of Structures A, B and C at various injection currents. The EQE is described by Equation S3 in Supplementary Note I. The maximum EQE for Structure B and Structure C are 1.6% and 2.5%, respectively; these values are 1.9 times and 2.8 times greater than those of Structure A.

The underlying physical mechanism of the improved emission performance for Structures B and C was elucidated via SiLENSe 5.14 (STR Software Inc.); this software can be used to calculate the energy band structure and carrier wavefunctions in the active region. In the simulation, the offset ratio between the conduction band and the valence band is set to 7:3^35^. The electron and hole mobilities are 100 and 10 cm^2^ V^−1^ s^−1^, respectively^36^. The Poisson equation, drift-diffusion transport equations, Fermi-Dirac statistics, and Schrödinger equations are used during the simulation. The Auger recombination coefficient, radiative recombination coefficient and Shockley-Read-Hall (SRH) recombination coefficient are set as 2.3 × 10^30^ cm^6^ s^−1^, 2 × 10^11^ cm^3^ s^−1^, and 1.1 × 10^7^ s^−1^, respectively^37–39^. Figure 2g–i illustrates the results of the calculated wavefunction overlap in the first QW region for Structures A, B and C at a bias of 5 V. As shown in Fig. 2g, the apparent separation resulting from the QCSE for carriers is observed in the first QW of Structure A (60%); this can degrade the radiative recombination between the holes and electrons. In contrast, the overlap ratios (*Γ*) of the holes and electrons in the first QW of Structures B and C increase from 60% (Structure A) to 68% (Structure B) and 70% (Structure C), as shown in Fig. 2h, i. This improvement can be ascribed to the alleviated QCSE, which originates from carrier localization by confining the holes and electrons in the QW. More detailed interpretations of the energy band and carrier recombination rate of Structures A, B and C are provided in Supplementary Note II.

### Employment of IS-SiO_2_ and its application in DUV LEDs

The p-GaN contact layer, together with the Ni/Au p-electrode, can strongly absorb the DUV photons emitted from the active region^40^, which can remarkably degrade the light extraction of DUV LEDs with staggered QW. Previous studies have shown that AlGaN TJ can remarkably suppress the absorption of DUV light and enhance the current injection efficiency of DUV LEDs^41–43^. To further improve the performance of DUV LEDs with staggered QW, we replace the absorbing p-GaN contact layer with a transparent AlGaN TJ and deposit an Al reflector on the Ni/Au as reflective p-electrode. The Structure C samples in Section 3.1 are used as the reference devices and are renamed LED I. The LED I samples with reflective p-electrodes are labeled as LED II. The LED II samples with AlGaN TJ are denoted as LED III. Supplementary Note III shows the schematic illustrations of the three kinds of DUV LEDs and discusses the corresponding issues on the emission performance enhancement of devices originating from the low-optical-loss ohmic contact layer and reflective p-electrode.

**Fig. 3:**
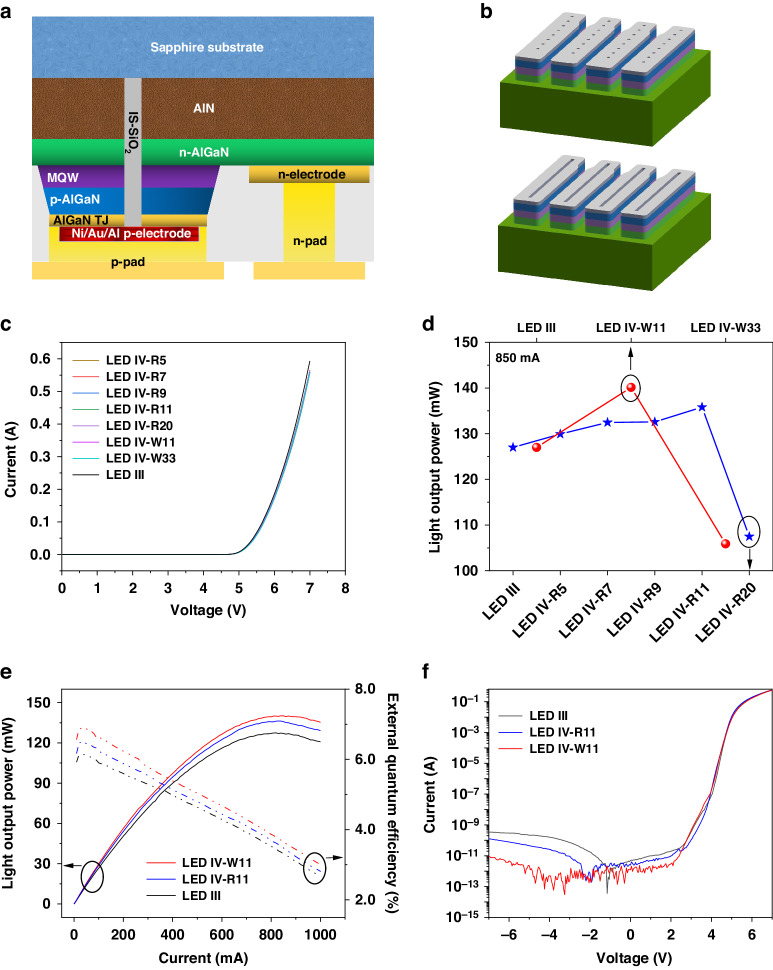
L-I-V characteristics of the DUV LEDs with IS-SiO_2_. Schematic illustration of (**a**) the DUV LED with IS-SiO_2_ and (**b**) the mesas with cuboid and columnar etching structures. **c** *I*‒*V* curves of LED III and LED IV. **d** Extractive LOP of LED III and LED IV at 850 mA. **e** LOP-I and EQE-I curves of LED III, LED IV-R11, and LED IV-W11. **f** Logarithmic *I-V* characteristics of LED III, LED IV-R11 and LED IV-W11

We investigated the impact of IS-SiO_2_ on the light extraction of DUV LEDs. The abovementioned LED III samples with a size of 30 × 30 mil^2^ are used as a reference. The device structure of the DUV LED with IS-SiO_2_ is illustrated in Fig. 3a. The typical fabrication process of a DUV LED with IS-SiO_2_ is discussed in Supplementary Note IV. The effects of size and shape of IS-SiO_2_ on DUV light extraction were investigated. The IS-SiO_2_ in DUV LEDs has two configurations: cuboid and columnar array, as shown in Fig. 3b. The cuboid IS-SiO_2_ has a fixed length of 425 μm and flexible widths of 11 and 33 μm. The columnar IS-SiO_2_ array is composed of eight single IS-SiO_2_ cylinders with flexible radii of 5, 7, 9, 11 and 20 μm. Both the cuboid and columnar IS-SiO_2_ configurations have the same height of ~6 μm. According to the size and shape of IS-SiO_2_, DUV LEDs with columnar IS-SiO_2_ are named LED IV-RX (X represents the radius of columnar IS-SiO_2_, X = 5, 7, 9, 11, or 20). The DUV LEDs with cuboid IS-SiO_2_ are named LED IV-WY (Y represents the width of cuboid IS-SiO_2_, *Y* = 11 or 33).

Figure 3c shows the measured *I*–*V* characteristics of the LED samples at room temperature. The tested DUV LEDs have almost the same turn-on voltage of ~5 V, indicating good consistency for the growth of DUV LEDs. As the voltage increases from 5 V, the tested DUV LEDs also show very similar *I*–*V* characteristics. Thus, IS-SiO_2_ will not increase the sheet resistance of the DUV LEDs, and the introduction of IS-SiO_2_ has a negligible influence on the electrical properties of the device. Figure 3d shows the extracted LOPs of the LED III and LED IV samples at 850 mA. The optimal sizes for both cuboid and columnar IS-SiO_2_ are found. For columnar IS-SiO_2_, the optimal radius is 11 μm. For cuboid IS-SiO_2_, the optimal width is 11 μm. Figure 3e shows the dependence of the LOPs on the injection currents for the LED IV-R11, LED IV-W11, and LED III samples. The LED III samples are used as the reference devices. The maximum LOP of the LED IV-W11 sample reaches 140.1 mW at 850 mA; this value is 4.3 and 12.8 mW greater than that of the LED IV-R11 and LED III samples, respectively. The peak EQEs of LED IV-W11, LED IV-R11, and LED III are determined to be 6.9%, 6.5% and 6.0%, respectively, by using Equation S3 in Supplementary Note I. Figure 3f shows the relationship between the reverse leakage current and voltage of the three DUV LEDs. At −5 V, the reverse leakage current is measured as 2.2 × 10^−10^, 4.5 × 10^−11^ and 2.3 × 10^−12 ^A for LED III, LED IV-R11, and LED IV-W11, respectively. The order of magnitude decrease in the reverse leakage current can be attributed to the screening of the channels for the leakage current originating from the introduction of the dielectric IS-SiO_2_. In addition, the screening effect of the continuous IS-SiO_2_ cuboid is clearly much stronger than that of the discrete IS-SiO_2_ cylinder. The LOP-I characteristics of LED IV with cuboid and columnar IS-SiO_2_ are shown in Fig. S5 (Supporting Information). A comparison of the emission performance of our DUV LEDs with that of previously reported DUV LEDs is shown in Table S1 and Fig. S6 (Supporting Information). The wafer-scale emission performances of DUV LEDs with cuboid and columnar IS-SiO_2_ are discussed in Supplementary Note IV.

**Fig. 4:**
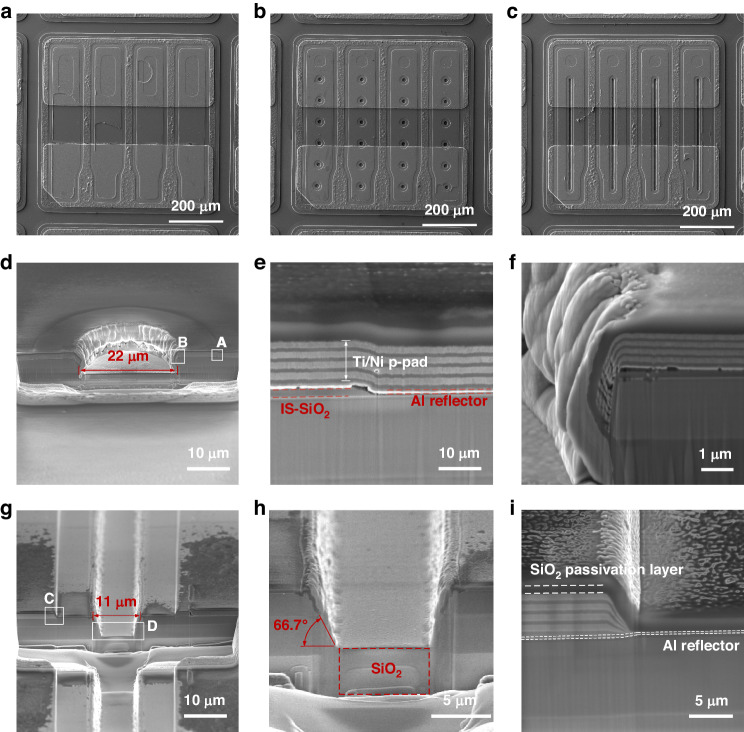
Morphology characteristics of the DUV LEDs with IS-SiO_2_. Top-view SEM images of (**a**) LED III, (**b**) LED IV-R11 and (**c**) LED IV-W11. **d** FIB-SEM image of LED IV-R11. Enlarged FIB-SEM images of Regions (**e**) A and (**f**) B in LED IV-R11. **g** FIB-SEM image of LED IV-W11. Enlarged FIB-SEM images of Regions (**h**) C and (**i**) D in LED IV-W11

The morphological characteristics of the DUV LEDs with a size of 30 × 30 mil^2^ containing IS-SiO_2_ are shown in Fig. 4. Figure 4a–c shows the top-view SEM images of LED III, LED IV-R11 and LED IV-W11. The LED samples with columnar and cuboid IS-SiO_2_ were sprayed with gold for FIB-SEM measurements. The tilt angle is 49° for the FIB-SEM measurements. Figure 4d–f shows the FIB-SEM images of LED IV-R11. As shown in Fig. 4d, the columnar etching structure is partly filled with SiO_2_; this represents a compromise between the design and fabrication of the LED device. The current IS-SiO_2_ is fabricated by combining photolithography and ICP etching, followed by PECVD. If the columnar etching structure is completely filled with SiO_2_, the residual SiO_2_ in Fig. 4e will be too thick to pose a problem for the deposition of the Ti/Ni p-pad. In addition, more etching is needed to remove the thick SiO_2_ residue; this is not only detrimental to the fabrication cost but also detrimental to the surface of the epitaxial wafer. The sidewall of the columnar etching structure is almost vertical in Fig. 4f, which is not beneficial for the deposition of SiO_2_, the Al reflector and the Ti/Ni/Ti/Ni p-pad. Figure 4g–i shows the FIB-SEM images of LED IV-W11. Additional photolithography and exposure for LED IV-W11 are performed to ensure that no Ti/Ni/Ti/Ni metal stacks are deposited on the etched groove, as shown in Fig. 4g. The depth of the etched groove is calculated to be ~3.7 μm. Unlike that of LED IV-R11, the etching facet of LED IV-W11 is sloping with an inclination angle of 66.7°, as shown in Fig. 4i. The slant etching facet is more favorable for the complete deposition of the IS-SiO_2_ and Al reflector, which strongly influences the current leakage and emission performance of the device.

### Light extraction analysis

To reveal the underlying mechanisms for the superior LOPs resulting from IS-SiO_2_ in the DUV LEDs, we further performed simulation experiments by using the SimuLED commercial software package and finite-difference time-domain (FDTD) method to investigate the influence of IS-SiO_2_ on the current distribution and light extraction of DUV LEDs. In all simulations, the refractive indices and absorption coefficients of AlGaN, MQWs, sapphire, and SiO_2_ were taken from the literature^44^.

We first simulated the current identity distribution in LED III, LED IV-R11 and LED IV-W11. The results from the simulated emission power densities of LED III, LED IV-R11, and LED IV-W11 at 350 mA and 850 mA are shown in Fig. S7 (Supporting Information). The calculated root mean square (RMS) values of the current density for LED III, LED IV-R11 and LED IV-W11 are 192.079, 198.315, and 202.172 A cm^−2^ at 350 mA, respectively. The RMS values of the current density for LED III, LED IV-R11, and LED IV-W11 are 500.731, 514.699, and 521.392 A cm^−2^ at 850 mA, respectively. Thus, insulated IS-SiO_2_ can enhance the effective current injected into the MQW and is anticipated to improve the LOP. However, the calculated output powers at 350/850 mA for LED III, LED IV-R11, and LED IV-W11 are 96.281/188.274, 95.553/186.774, and 95.102/186.143 mW, respectively. These paradoxical results can be ascribed to the dual character of IS-SiO_2_. On the one hand, IS-SiO_2_ can enhance the effective current injection to increase the emission power. On the other hand, the introduction of IS-SiO_2_ leads to an area loss of the MQW structure. The positive and negative effects of IS-SiO_2_ provide a balance and produce in similar output powers for the three LED samples.

Since the current distribution simulation is not sufficient to elucidate the improvement in LOP resulting from IS-SiO_2_, we employed FDTD simulation to investigate the light extraction behavior inside the devices. To simplify the computations, the sizes of all LED simulation models need to be scaled down. The sizes of the LED simulation models are shown in Fig. S8 (Supporting Information). First, we simulated the electrical field intensity distribution of LED III and LED IV-W11, as shown in Fig. S9 (Supporting Information). The total LEE of LED IV-W11 (32.0%) is greater than that of LED III (30.9%). The top LEE of LED IV-W11 (9.5%) is also greater than that of LED III (8.4%). We speculate that IS-SiO_2_ can break the TM-polarized waveguide of DUV light and simultaneously enhance the transverse-electric (TE)-polarized waveguide, thereby increasing the LEE of the top facet. However, the electrical field intensity distribution of LED IV-W11 did not sufficiently improve the LEE of the top facet. Thus, the sapphire substrate is potentially too thick to show the effect of IS-SiO_2_ on the light extraction of the top facet.

**Fig. 5:**
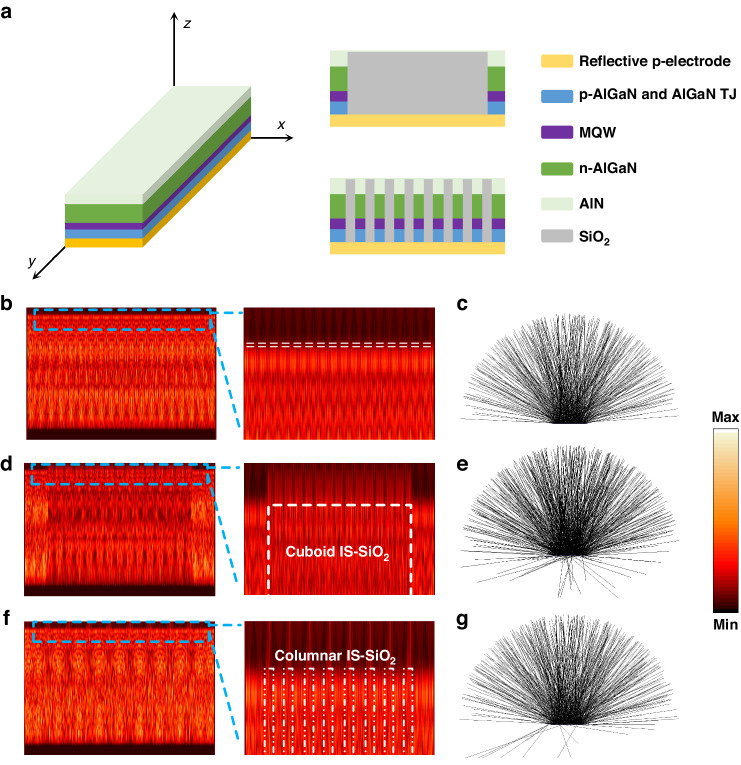
Simulations for the light extraction behaviors of the DUV LEDs with IS-SiO_2_ based on FDTD and Monte Carlo ray-tracing methods. **a** Schematic illustration of the simulation models for LED IV-W11 and LED IV-R11. The radius of the columnar IS-SiO_2_ is set as 1.1 μm. The *x* plane of the normalized electrical field intensity distribution of (**b**) LED III, (**d**) LED IV-W11, and (**f**) LED IV-R11. Cross-sectional ray-tracing images of (**c**) LED III, (**e**) LED IV-W11, and (**g**) LED IV-R11

To verify the abovementioned deduction regarding IS-SiO_2_, we simplified the simulated models of LED III, LED IV-R11 and LED IV-W11 by removing the sapphire substrate, as shown in Fig. 5a. The normalized electrical field intensity distributions of LED III, LED IV-W11 and LED IV-R11 are shown in Fig. 5b, d, f. For LED IV-R11 and LED IV-W11, the electrical intensity field distributions on the surface of the simulation models are both much stronger than those of LED III. In addition, the electrical intensity field distributions on the surfaces of LED IV-R11 and LED IV-W11 are discrete and continuous; this results are in agreement with the arrangements of the corresponding IS-SiO_2_. The Monte Carlo ray-tracing results of LED III, LED IV-R11 and LED IV-W11 in Fig. 5c, e, g are used to further verify the simulation results from the FDTD methods.

**Fig. 6:**
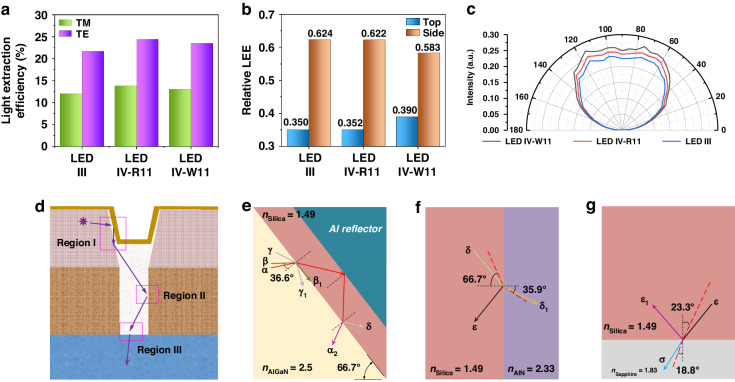
Analyses of the light extraction behaviors of the DUV LEDs with IS-SiO_2_. **a** TE-/TM-polarized LEEs, **b** relative LEEs at the top/side facets, and **c** simulated far-field radiation patterns of LED III, LED IV-R11 and LED IV-W11. **d** Schematic illustration of light trajectories inside the IS-SiO_2_ of LED IV-W11. Analyses of light trajectories in Regions (**e**) I, (**f**) II and (**g**) III

Moreover, we further quantified the improvement in TE-/TM-polarized light extraction achieved by introducing IS-SiO_2_. Figure 6a shows the calculated TE- and TM-polarized LEEs of LED III, LED IV-R11 and LED IV-W11. The TE-/TM-polarized LEEs of LED III, LED IV-R11 and LED IV-W11 are 21.7/12.7%, 24.5/13.8% and 23.5/13.1%, respectively. Compared with the TE-/TM-polarized LEEs of LED III, those of LED IV-R11 and LED IV-W11 are increased by 2.8/1.1% and 1.8/0.4%, respectively. Therefore, in comparison to that of LED III, the LEE enhancement of TE-/TM-polarized light for LED IV can be primarily attributed to IS-SiO_2_. Then, we calculated the relative LEE (*φ*_r_) for the three LED samples by using Eqs. (1) and (2):

1 $${{\rm{\varphi }}}_{{\rm{r}},{\rm{sides}}}{\boldsymbol{=}}\frac{{{\rm{\varphi }}}_{{\rm{TE}},{\rm{sides}}}+{{\rm{\varphi }}}_{{\rm{TM}},{\rm{sides}}}}{{{\rm{\varphi }}}_{{\rm{TE}}}+{{\rm{\varphi }}}_{{\rm{TM}}}}$$

2 $${{\rm{\varphi }}}_{{\rm{r}},{\rm{top}}}{\boldsymbol{=}}\frac{{{\rm{\varphi }}}_{{\rm{TE}},{\rm{top}}}+{{\rm{\varphi }}}_{{\rm{TM}},{\rm{top}}}}{{{\rm{\varphi }}}_{{\rm{TE}}}+{{\rm{\varphi }}}_{{\rm{TM}}}}$$

where *φ*_r, sides_ and *φ*_r, top_ are the relative LEE of the side and top facets for the LED sample, respectively, *φ*_TE_ and *φ*_TM_ are the LEE of the TE- and TM-polarized light, respectively, *φ*_TE, sides_ and *φ*_TM, sides_ are the LEE of the TE- and TM-polarized light emitted from the side facets of the LED chip, respectively, and *φ*_TE, top_ and *φ*_TM, top_ are the LEE of the TE- and TM-polarized light emitted from the top facet of the LED chip, respectively. Based on Equations (1) and (2), the calculated *φ*_r, sides_ and *φ*_r, top_ of LED III, LED IV-R11 and LED IV-W11 are shown in Fig. 6b. Clearly, reduced sidewall emission and enhanced top emission are achieved by the IS-SiO_2_ in the LED IV. Figure 6c shows the simulated far-field radiation patterns of LED III, LED IV-R11 and LED IV-W11. The estimated LEEs for LED III, LED IV-R11, and LED IV-W11 are 4.47%, 4.97% and 5.45%, respectively. These results are consistent with those of other simulations and experiments and further confirm the superior light extraction ability of IS-SiO_2_.

To understand the physical mechanism of the improved LEE at the top facet of the DUV LED with IS-SiO_2_, the light trajectories at different interfaces are analyzed using Snell’s law. Figure 6d shows the typical light trajectory inside the IS-SiO_2_ of LED IV-W11. We only focus on the light rays that travel in the IS-SiO_2_ before leaving the SiO_2_. Figure 6e shows the light trajectory analysis in Region I. According to the refractive indices of AlGaN and SiO_2_, the critical angle of total internal reflection at the AlGaN/SiO_2_ interface is 36.6°, such as light ray **β**. When the incident angle is *θ* < 36.6°, the light rays radiating on the AlGaN/SiO_2_ interface are refracted into IS-SiO_2_, such as light ray **α**. If 36.6° < *θ* < 90°, the light rays are reflected back to the AlGaN epitaxial layer, such as light ray **γ**. Based on the device design and light trajectory diagnosis, light rays **β** and **γ** are basically emitted from the top facet of the flip-chip DUV LED. The light ray **α** is reflected back to the AlGaN/SiO_2_ interface by the reflective p-electrode, followed by further reflection and refraction in the IS-SiO_2_. The refractive light ray, the light ray **α**_**1,**_ is transmitted in the AlGaN epitaxial layer. Light ray **δ** (the reflective light ray) repeats the transmission route of light ray **α** and finally arrives at Region II after experiencing repetitive oscillations in IS-SiO_2_. Figure 6f shows the typical light trajectories at the AlN/IS-SiO_2_ interface in Region II. The incident light ray from Region I (light ray **δ**) is reflected and refracted at the AlN/IS-SiO_2_ interface, thereby resulting in the generation of the light ray **δ**_**1**_ (refractive light ray) and the light ray **ε** (reflective light ray). The incident angle of the light ray **δ** cannot exceed 66.7°; this angle is the incline angle of the etching facet in Fig. 6e. Correspondingly, the maximum refraction angle of the light ray **δ**_**1**_ is 35.9°. The light ray **ε** enters Region III as the incident light in Fig. 6g. Light ray **ε** is reflected and refracted at the IS-SiO_2_/Al_2_O_3_ interface, thus leading to the formation of light ray **ε**_**1**_ and the light ray **σ**. Light ray **σ** is emitted from the bottom of IS-SiO_2_ with a refraction angle larger than 18.8°. Light ray **ε**_**1**_ is reflected into the IS-SiO_2_ with a reflection angle larger than 23.3°.

## Conclusion

In summary, the integration of the tailored MQWs, a low-optical-loss p-electrode/ohmic contact layer and insulating IS-SiO_2_ is achieved in our DUV LEDs; these devices deliver an LOP of 140.1 mW at 850 mA. More importantly, the EQEs of our DUV LEDs are enhanced by 4.5 times in contrast to those of their conventional counterparts. Moreover, to validate the experimental results, the band structure, current distribution and FDTD simulations were used to investigate the carrier radiative recombination and light extraction behavior of our DUV LEDs. Furthermore, the on-wafer electroluminescence properties of our DUV LEDs confirm that our method can be expanded to mass manufacturing scales. The understanding and exploitation of bandgap engineering and device craft presented in this study provide a widely applicable strategy for fabricating high-power AlGaN-based DUV emitters for use in biomedical testing, water/air purification, and other relevant fields.

### Supplementary information

Supplemental Material
