# Supplementary material for: Highly efficient AlGaN-based deep-ultraviolet light-emitting diodes: from bandgap engineering to device craft
Source: Microsyst Nanoeng. 2024 Aug 13;10:110. doi: 10.1038/s41378-024-00737-x (PMC11322536; doi:10.1038/s41378-024-00737-x)
Supplement: Supplementary file 1 — Supplemental Material [file 41378_2024_737_MOESM1_ESM.docx]

**Supporting Information**

**Highly Efficient AlGaN-based Deep-ultraviolet Light-emitting Diodes: from Bandgap Engineering to Device Craft**

Xu Liu^1,#^, Zhenxing Lv^1,#^, Zhefu Liao^1^, Yuechang Sun^1^, Ziqi Zhang^1^, Ke Sun^1^, Qianxi Zhou^1^, Bin Tang^1^, Hansong Geng^3^, Shengli Qi^3^ and Shengjun Zhou^1,2,&^

^1^Center for Photonics and Semiconductors, School of Power and Mechanical Engineering, Wuhan University, Wuhan 430072, China

^2^The Institute of Technological Sciences, Wuhan University, Wuhan, 430072, China

^3^Ningbo ANN Semiconductor Co., Ltd., Ningbo 315336, China

^#^These authors contributed equally to this work.

^&^Correspondence Email: zhousj@whu.edu.cn

**This Supplementary Information file includes:**

1. ***Fig. S1-S16***
2. ***Supplementary Note I~IV***
3. ***Table S1~S6***
4. ***Equation S1~S3***


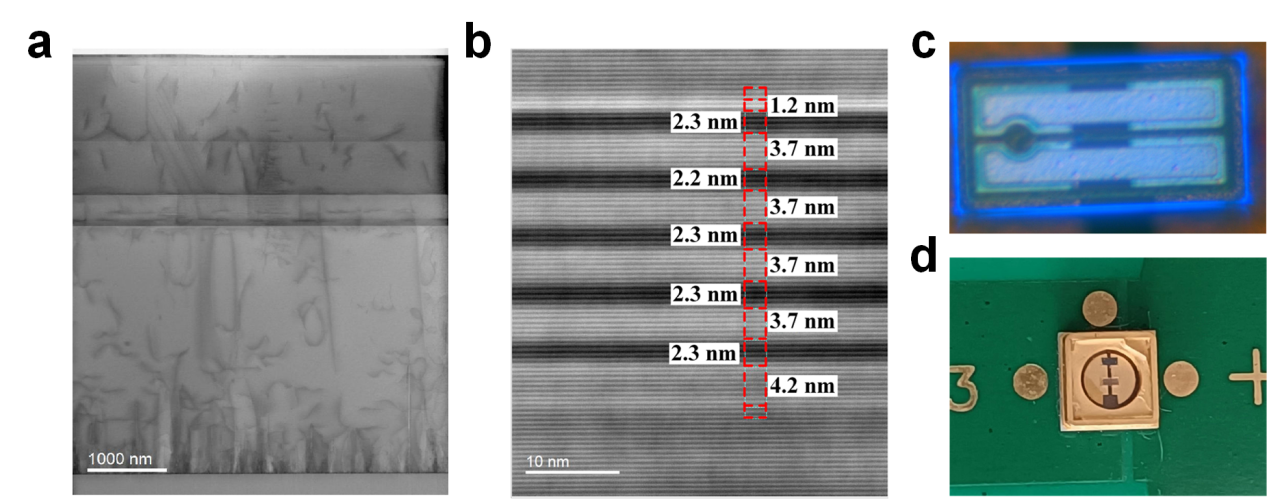


Fig. S1. (a) Cross-section SEM image of AlGaN-based epitaxial wafer. (b) Cross-section TEM image of corresponding MQW in the epitaxial wafer. (c) [Optical microscope](javascript:;) image of a lighted DUV LED. (d) P[hotograph](https://cn.bing.com/dict/search?q=photograph&FORM=BDVSP6&cc=cn) of a packaged DUV LED on the printed circuit board.


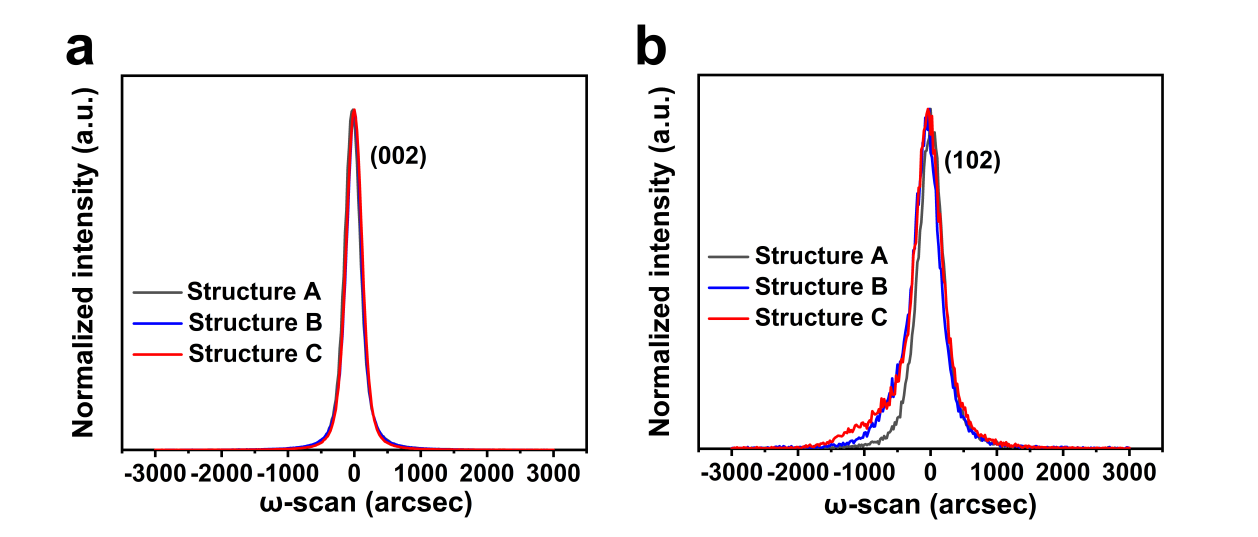


Fig. S2. (a) Symmetric (002) and (b) asymmetric (102) XRD rocking curves of Structure A, B and C.


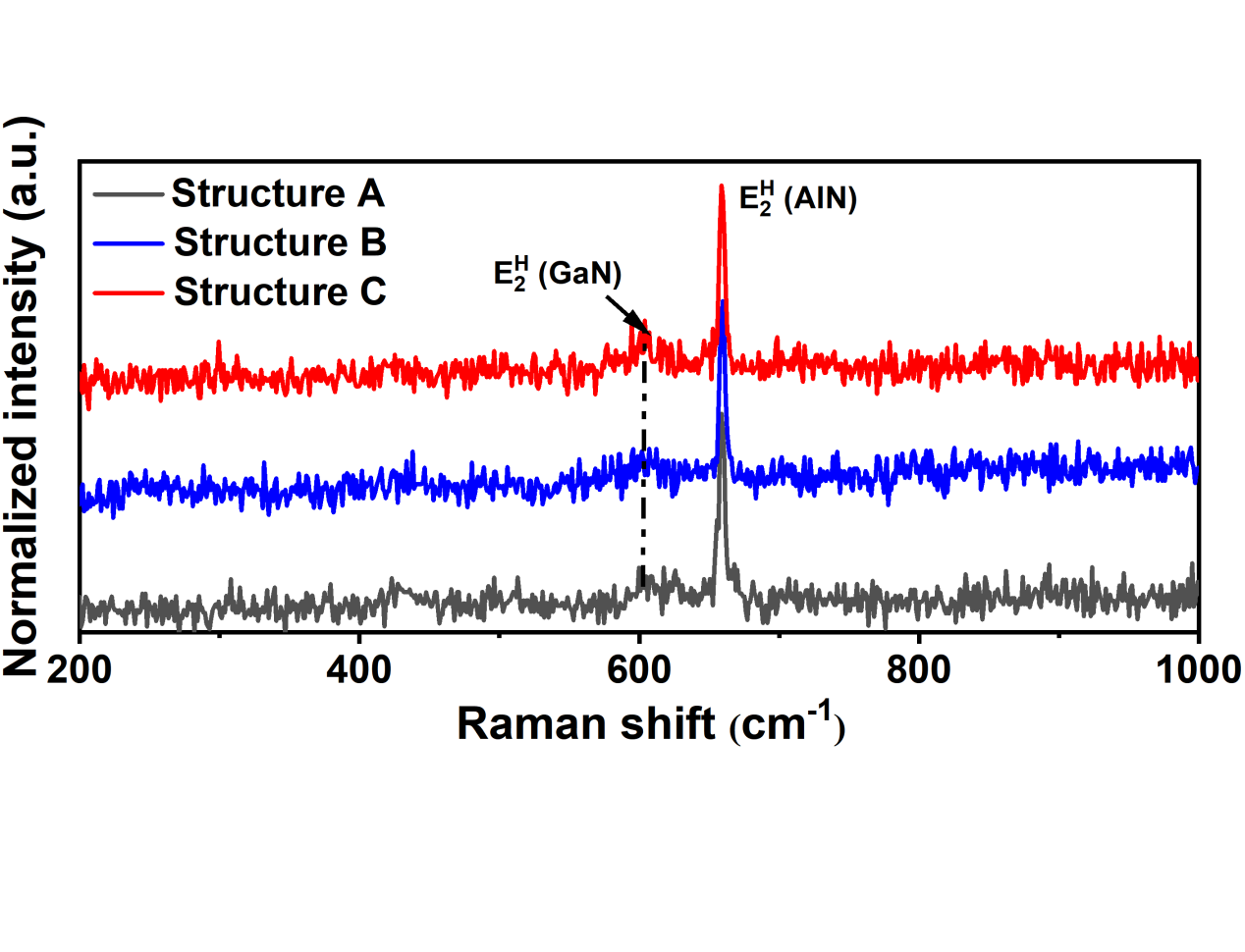


Fig. S3. Raman spectra of Structure A, B and C at room temperature.

***
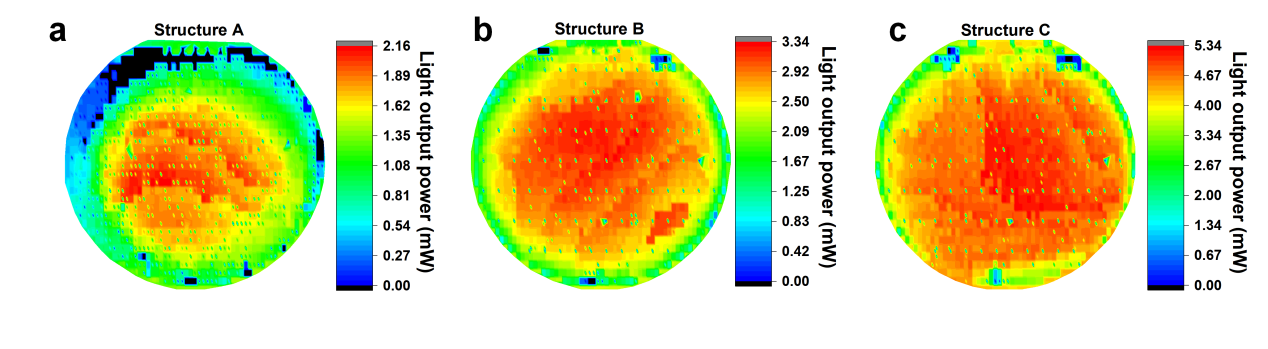
***

Fig. S4. Wafer-scaled emission performances of (a) Structure A, (b) Structure B, and (c) Structure C at 40 mA. The chips used to test the L-V-I characteristics are selected from the region in red color.


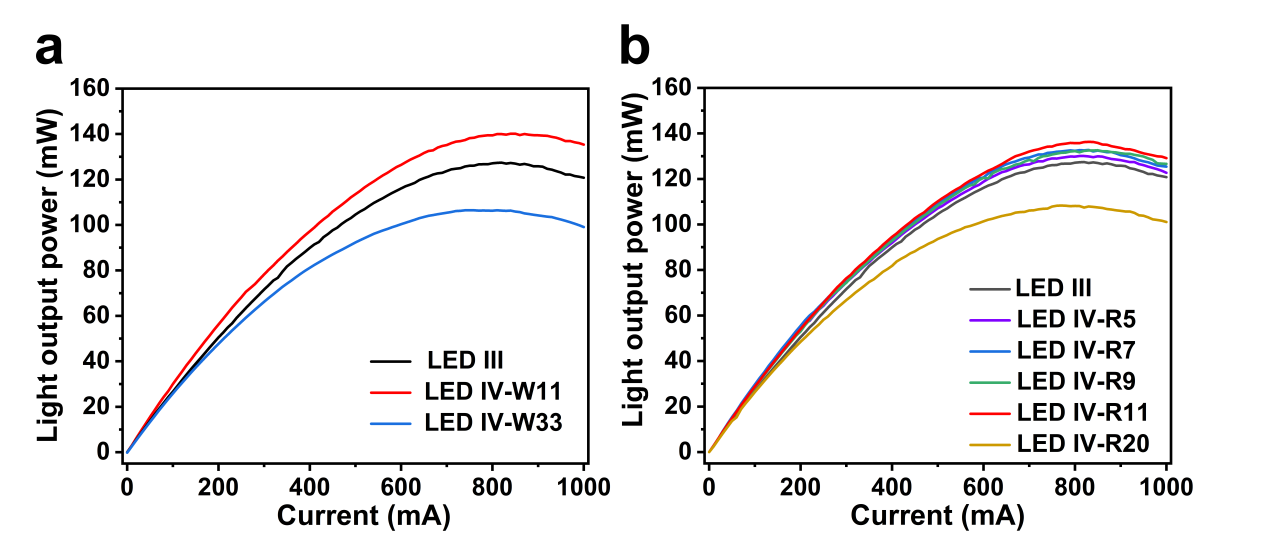


Fig. S5. LOP-I curves of the LED IV samples with (a) cuboid and (b) columnar IS-SiO_2_.


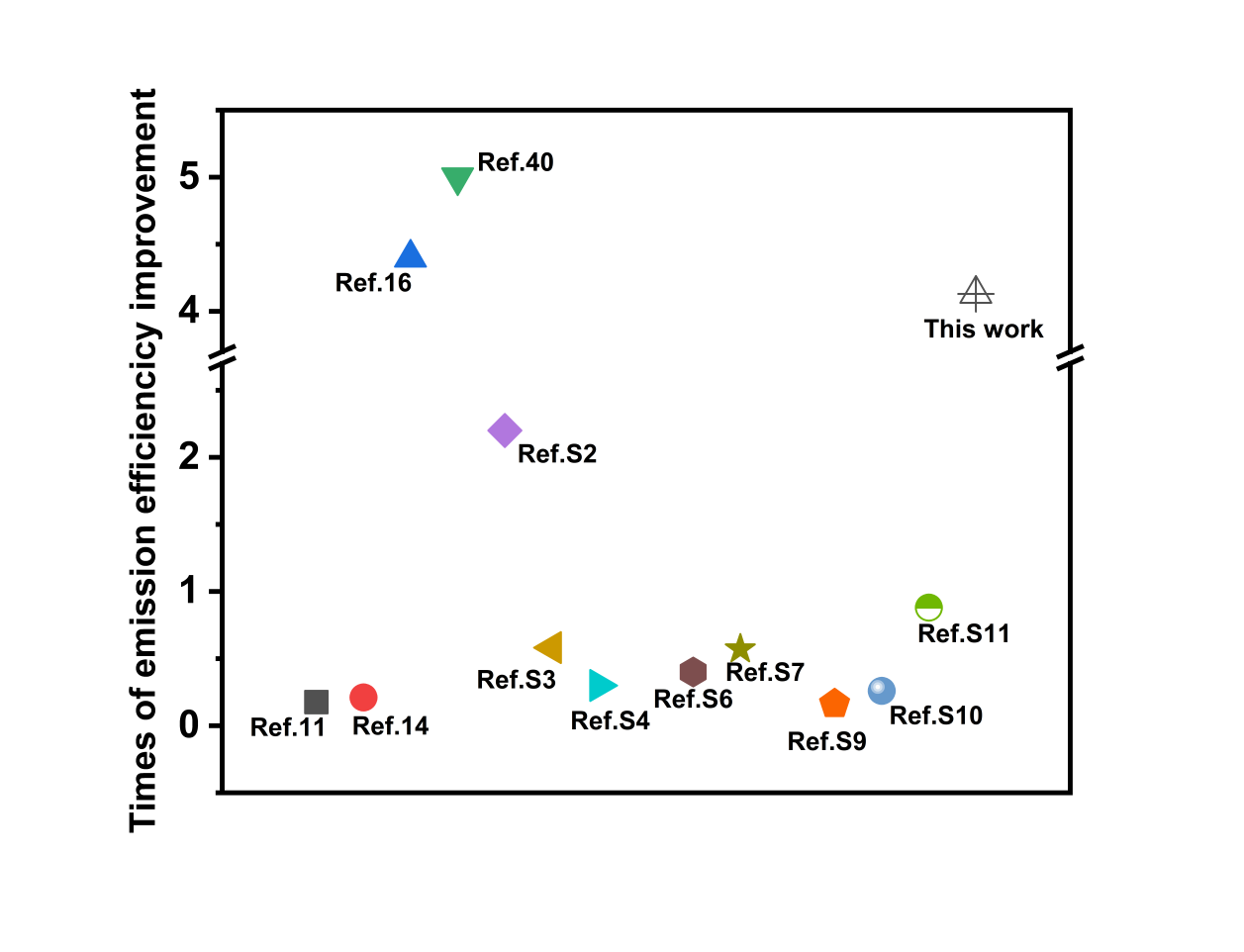


Fig. S6. Comparison of emission efficiency improvement of the our DUV LEDs with reported ones.

| Table S1. Comparison of DUV LEDs based on traditional and innovative configuration reported previously. | | | | | |
| --- | --- | --- | --- | --- | --- |
|  | **Wavelength**  **(nm)** | **Peak LOP**  **(mW)** | **EQE/WPE**  **(%)** | **Device Size**  **(μm^2^)** | **Reference** |
| **DUV LED**  **with NPSS/reflector** | ~275 | 25  (150 mA) | 4.7  (EQE) | 508×508 | Ref.11 |
| **Ni-sacrificed**  **DUV LED** | 275 | ~7  (100 mA) | 1.73  (EQE) | 1000×1000 | Ref.14 |
| **DUV LED**  **with Optimized MQW** | 275 | ~11  (220 mA) | ~2.1  (EQE) | 305×508 | Ref.16 |
| **AlGaN**  **DUV LED** | 275 | 44.2  (50 mA) | 20  (EQE) | 500×500 | Ref.40 |
| **AlGaN**  **DUV LED** | 285 | ~6  (30 mA) | 10.6  (EQE) | 100×100 | Ref.S1 |
| **DUV LED**  **with Optimized p-layers** | 275 | 385  (1500 mA) | 15.7  (EQE) | 1000×1000 | Ref.S2 |
| **TJ-AlGaN**  **DUV LED** | 265 | - | 11  (EQE) | 40×40 | Ref.S3 |
| **DUV LED with Microcavities** | 260 | - | - | 400×800 | Ref.S4 |
| **Ring-shaped**  **DUV LED** | ~280 nm | 53.36 W/cm^2^  (650 A/cm^2^) | 6.17  (EQE) | 90^2^π | Ref.S5 |
| **AlGaN**  **DUV LED** | 277 | 15.1  (100 mA) | 3.5  (EQE) | 510×510 | Ref.S6 |
| **AlGaN**  **DUV LED** | 278 | 15.9  (100 mA) | 3.3  (WPE) | 510×510 | Ref.S7 |
| **Core−shell Nanowire** | 275 | 8  (220 A/cm^2^) | 0.4  (EQE) | 1000×1000 | Ref.S8 |
| **Plasmon-enhanced**  **μ-LED Array** | 275 | ~8.0  (67 A/cm^2^) | ~3.3  (EQE) | 1130×950 | Ref.S9 |
| **μ-LED** | 277 | 0.19  (3000 A/cm^2^) | 0.225  (EQE) | 700 | Ref.S10 |
| **Parallel-arrayed**  **Planar μ-LED** | ~280 | 83.5  (230 mA) | 4.7  (WPE) | 125×125 | Ref.S11 |
| **Hybrid**  **DUV LED** | 281 | 0.033  (1.87 mA) | 21.6  (WPE) | 762×762 | Ref.S12 |
| **This work** | 270 | 140.1  (850 mA) | 6.9  (EQE) | 762×762 | - |


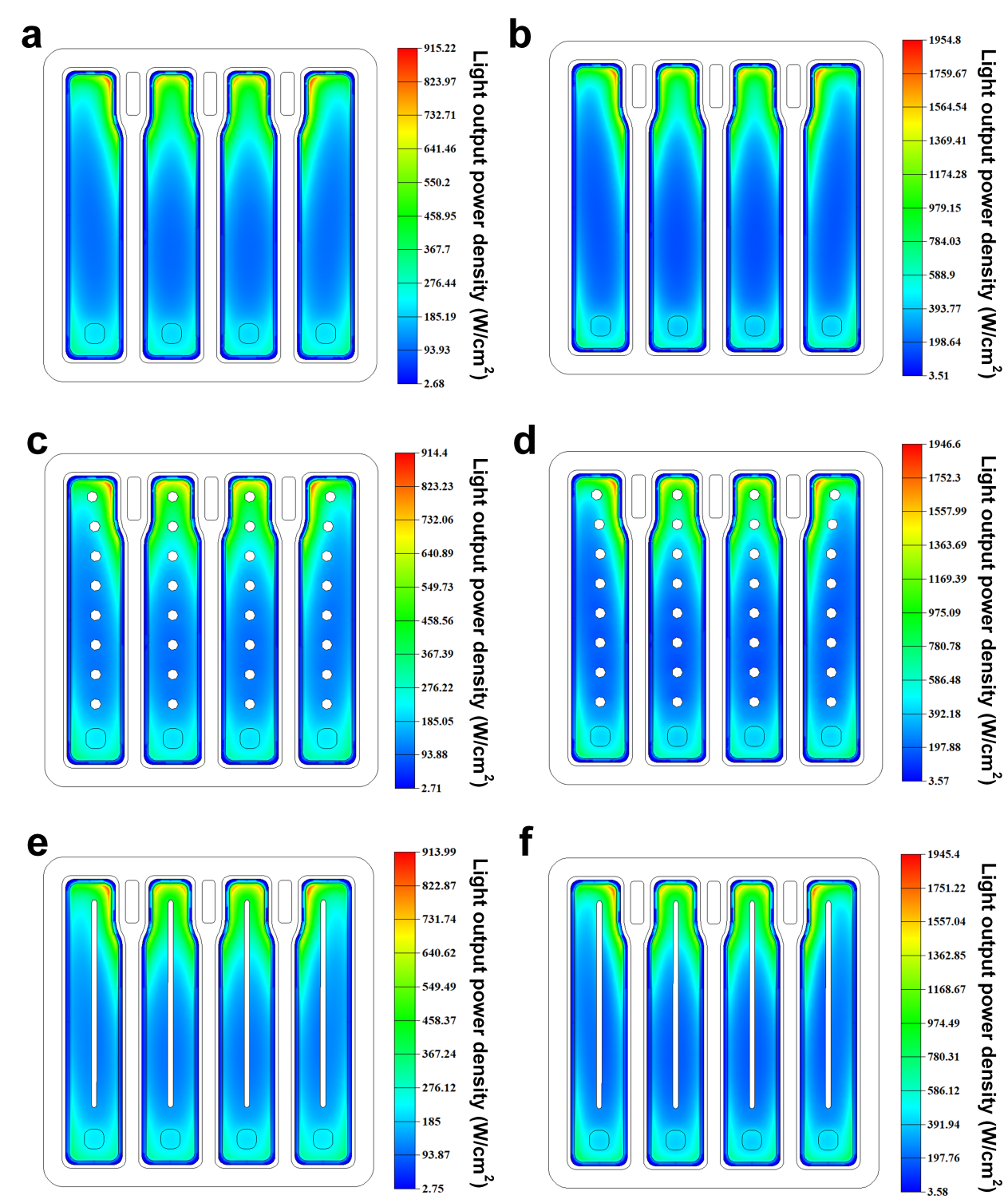


Fig. S7. Calculated output power density of (a) LED III, (c) LED IV-R11 and (e) LED IV-W11 at 350 mA. Calculated output power density of (b) LED III, (d) LED IV-R11 and (f) LED IV-W11 at 850 mA. All the output power density are calculated by using SimuLED commercial software package.


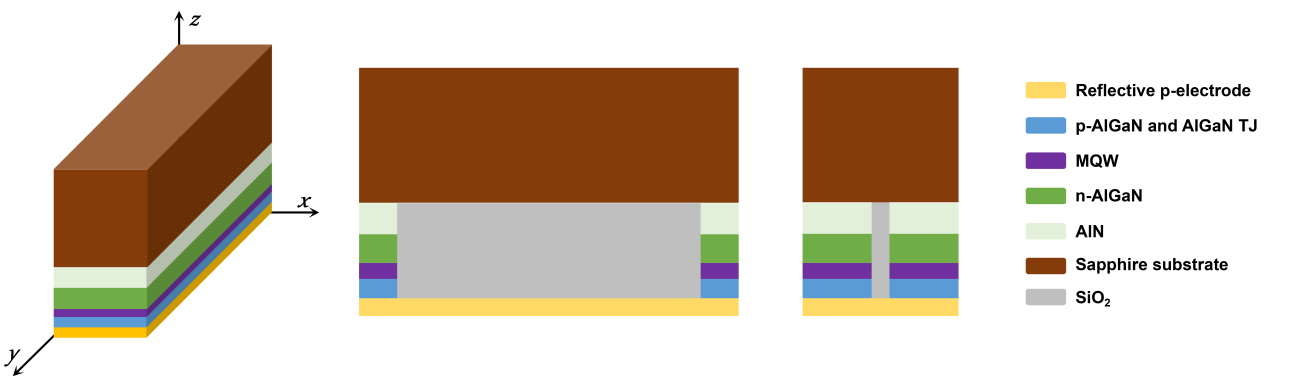


Fig. S8. Schematic illustration of entire simulation model for LED IV-W11. The width, length and thickness of simulation model for LED IV-W11 is set as 55.9, 9.025 and 14.7 μm, respectively. The width, length and thickness of simulation model for cuboid IS-SiO_2_ is set as 1.1, 42.5 and 1.4 μm, respectively.


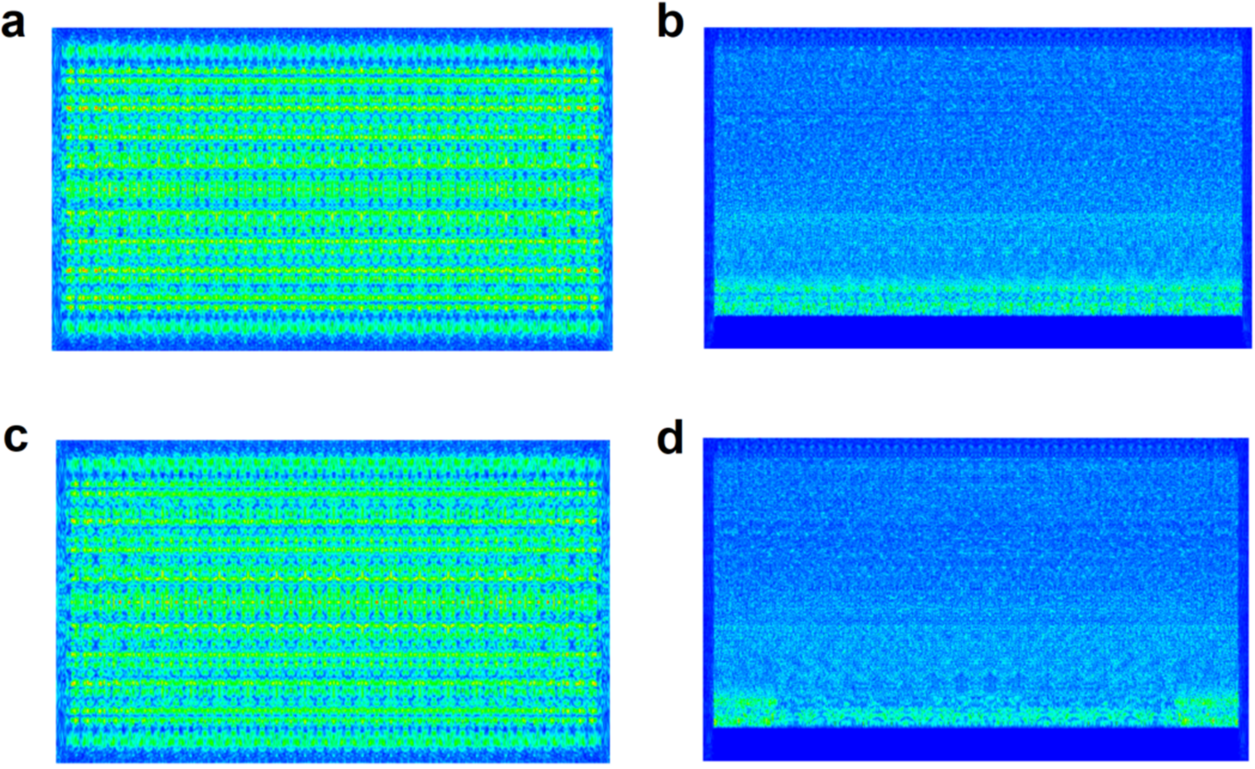


Fig. S9. The *z* plane of normalized electrical field intensity distribution of (a) LED III and (c) LED IV-W11. The *x* plane of normalized electrical field intensity distribution of (c) LED III and (d) LED IV-W11.

***Supplementary Note I***

**Equation S1**:

$$\begin{aligned} \boldsymbol{N=}\frac{\boldsymbol{\beta}^{\boldsymbol{2}}}{\boldsymbol{4.36\times}\left| \boldsymbol{b} \right|^{\boldsymbol{2}}} \end{aligned}$$

where ***N*** is the dislocation density, ***b*** is the magnitude of the Burgers vector, and ***β*** is the FWHM of the XRD rocking curve.

**Equation S2^13^**:

${\boldsymbol{k}\boldsymbol{\sigma}}_{\boldsymbol{xx}}\boldsymbol{=}\boldsymbol{\Delta}$***ω***

where $\boldsymbol{\Delta}$***ω*** is the the Raman shift of the E_2_^H^ peak in respect to reference, ***k*** is the biaxial strain coefficient, 3.7 cm^-1^/GPa.

**Equation S3**:

$$\begin{aligned} \boldsymbol{\eta}_{\boldsymbol{EQE}}\boldsymbol{=}\frac{{\boldsymbol{P}_{\boldsymbol{out}}}/\boldsymbol{h\nu}}{{\boldsymbol{I}_{\boldsymbol{in}}}/\boldsymbol{e}}\boldsymbol{=}\frac{\boldsymbol{P}_{\boldsymbol{out}}\boldsymbol{\lambda}}{\boldsymbol{1240}\boldsymbol{I}_{\boldsymbol{in}}} \end{aligned}$$

where ***P_out_*** is the LOP of the DUV LEDs, ***λ*** is the wavelength of emission light, ***I_in_*** is the injection current, ***e*** is the elementary charge, ***h*** is the Planck constant, ***ν*** is the frequency of DUV light emitted from MQWs.

| Table S2. Summaries on the abbreviations of DUV LED samples. | | | |
| --- | --- | --- | --- |
| **Sample** | **Note** | **Chip size** | **Location** |
| **Structure A** | DUV LED originated from the wafer with conventional QWs | 10 × 20 mil^2^ | Section 3.1 |
| **Structure B** | DUV LED originated from the wafer with gradient QWs |  |  |
| **Structure C** | DUV LED originated from the wafer with staggered QWs |  |  |
| **LED I** | DUV LED originated from the wafer with staggered QWs, reference sample | 30 × 30 mil^2^ | Supplementary Note III |
| **LED II** | LED I with reflective p-electrode |  |  |
| **LED III** | LED II with AlGaN TJ |  |  |
| **LED IV-RX** | LED III with columnar IS-SiO_2_. X presents the radius of columnar IS-SiO_2_ (X=5, 7, 9, 11 and 20) | 30 × 30 mil^2^ | Section 3.2 |
| **LED IV-WY** | LED III with cuboid IS-SiO_2_. Y presents the width of cuboid IS-SiO_2_ (Y=11 and 33). |  |  |

***Supplementary Note II***


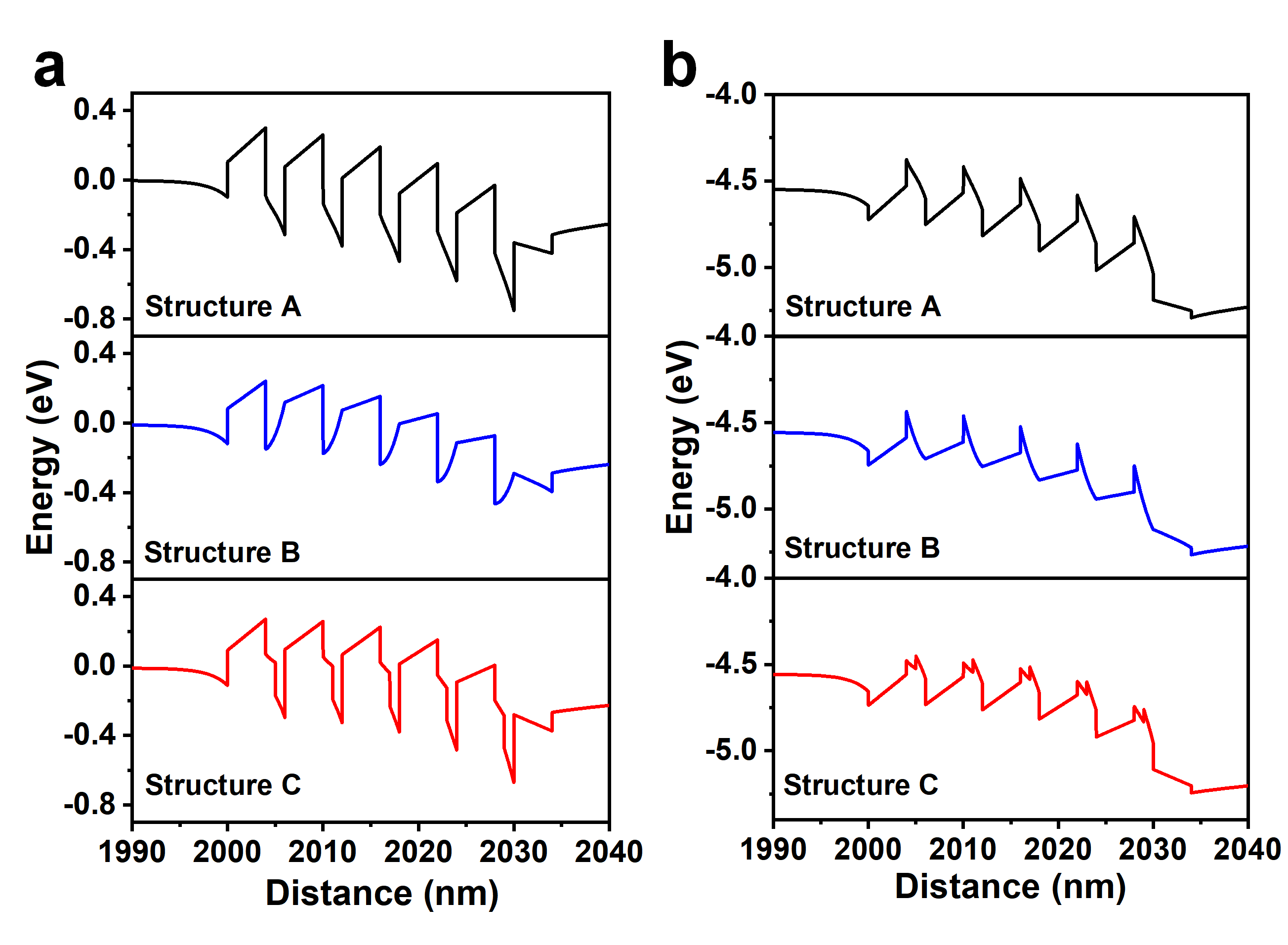


Fig. S10. Enlarged (a) conduction and (b) valence band diagrams in the MQW of Structure A, B and C at 5 V.

The calculated conduction and valence band diagrams of Structures A, B and C are shown in **Fig. S10(a-b)**. The energy band is tilted by the polarization-induced electric field, thus resulting in accumulation of carriers opposite sides of QWs. It will make carrier wave-function separated, thus deteriorating the emission performance of LED chips. The polarization-induced electric field makes the energy band tilted, leading to carrier accumulation in opposite sides of QWs and a spatial separation of carrier wave function. As shown in **Fig. S10(a)**, the electron-effective barrier height of Structures A, B and C is 616, 134 and 465 meV, respectively. It is illustrated that the electron blocking ability of Structure A is better than those of Structure B and C. In **Fig. S10(b)**, the effective barrier height of Structures A, B and C for holes is 330, 260 and 250 meV, respectively. The effective barrier height for holes of Structure B is slightly higher than that of sample C. It can be concluded that MQW structure in Structure C is more beneficial for hole injection than Structures B and A. Compared to Structure A, the lower hole-effective barrier height makes more holes injected into the MQW structure of Structure C. In addition, the enough high electron-effective barrier height can guarantee that there are abundant electrons retained in the MQW of Structure C, thereby adequately consuming injected holes via radiative recombination. Structure C has slightly lower hole- and much higher electron-barrier height than Structure B. The almost same hole-barrier heights of Structures B and C guarantees that hole concentration in the MQWs of Structure B and C are the identical to a first approximation. However, the lower electron-barrier height in the MQW of Structure B cannot entrap enough electrons for radiative recombination with holes in comparison to Structure C. In addition, the ultra-thin quantum barriers in the MQW of the three DUV LED samples facilitate the intraband tunneling process for carriers, which further decreases the possibility of radiative recombination between holes and electrons in the Structure B to some extent. Therefore, Structure C with the staggered MQW structure can be more favorable for radiative recombination than Structure A and B, which is consistent with the measured L-I characterization in **Fig. 2(e)**.


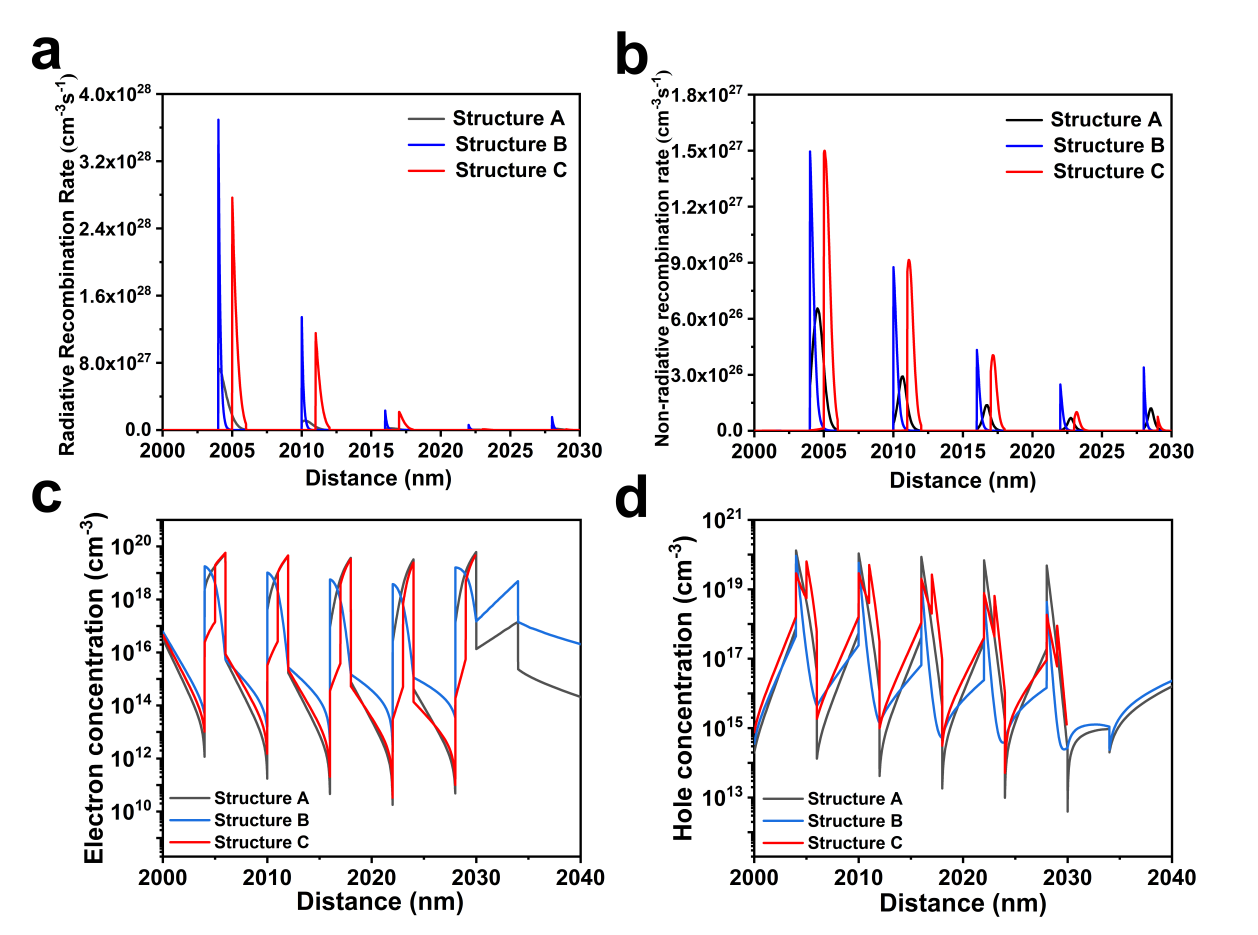


Fig. S11. (a) Radiative and (b) non-radiative recombination rate of carriers in the QWs of Structures A, B and C at 5 V. (c) Electron and (d) hole concentration in the MQWs of of Structures A, B and C at 5 V.

**Fig. S11(a-b)** show the calculated radiative and non-radiative recombination rates in the MQWs of Structures A, B and C. It is clear the first QWs near the n-AlGaN of the three samples are the major regions for the recombination behaviour of carriers. In the first QW, Structures B and C both exhibit the outstanding peak radiative recombination rates, which is almost 5.1- (Structure B) and 3.8-fold (Structure C) higher the that of Structure A, respectively, as shown in **Fig. S11(a)**. In contrast, the peak non-radiative recombination rates of Structures B and C are both 1.9-flod higher than that of Structure A in **Fig. S11(b)**. The i[ntegral](https://cn.bing.com/dict/search?q=Integral&FORM=BDVSP6&cc=cn) [area](https://cn.bing.com/dict/search?q=Area&FORM=BDVSP6&cc=cn) of radiative and non-radiative recombination rate curves can be used to calculate the total number of carriers participating in the recombination per [unit](https://cn.bing.com/dict/search?q=unit&FORM=BDVSP6&cc=cn) [time](https://cn.bing.com/dict/search?q=time&FORM=BDVSP6&cc=cn), as shown in **Table S3**. In contrast to Structure A, the carriers participating in the radiative recombination are increasing by 12.1% in the MQW of Structure B. Meanwhile, the carriers consumed by the non-radiative recombination in the MQW of Structure B are cut down by 11.9 %. The carriers for non-radiative recombination in the MQW of Structure C are much more than those in the MQW of Structure B. However, the carriers for radiative recombination in the MQW of Structure C are almost two times more than that in the MQW of Structure B, which is proved by the LOP-I characteristics of Structures A, B and C in **Fig. 2(e).** In addition, we also calculated the electron and hole concentration in the MQWs of Structures A, B and C, as shown in **Fig. S11(c-d)**. The i[ntegral](https://cn.bing.com/dict/search?q=Integral&FORM=BDVSP6&cc=cn) [area](https://cn.bing.com/dict/search?q=Area&FORM=BDVSP6&cc=cn) of electron and hole concentration curves can be used to calculate the sums of carriers in the MQWs of Structures A, B and C. The sums of electrons for Structures A, B and C are 1.43 × 10^20^, 4.81 × 10^19^ and 1.06 × 10^20^, respectively. The sums of holes for Structures A, B and C are 1.09 × 10^20^, 2.69 × 10^19^ and 7.30 × 10^20^, respectively. All the calculated results are shown in **Table S4**. Obviously, the tailored QWs in Structures B and C can improve the emission performance of devices though enhancing the radiative recombination efficiency of electron-hole pairs [rather](https://cn.bing.com/dict/search?q=rather&FORM=BDVSP6&cc=cn) [than](https://cn.bing.com/dict/search?q=than&FORM=BDVSP6&cc=cn) increasing the amounts of carriers injected into the active regions in contrast to the conventional QWs in Structure A.

| Table S3. Sums of electron-hole pairs participating in the recombination per [unit](https://cn.bing.com/dict/search?q=unit&FORM=BDVSP6&cc=cn) [time](https://cn.bing.com/dict/search?q=time&FORM=BDVSP6&cc=cn) in the MQW of Structures A, B and C. | | | |
| --- | --- | --- | --- |
|  | **Structure A** | **Structure B** | **Structure C** |
| **Radiative Recombination** | 6.76 × 10^27^ | 7.58 × 10^27^ | 1.42 × 10^28^ |
| **Non-radiative Recombination** | 1.04 × 10^27^ | 9.16 × 10^26^ | 1.47 × 10^27^ |

| Table S4. Calculated sums of carriers in the MQW of Structures A, B and C. | | | |
| --- | --- | --- | --- |
| **Carriers** | **Structure A** | **Structure B** | **Structure C** |
| **Electron** | 1.43 × 10^20^ | 4.81 × 10^19^ | 1.06 × 10^20^ |
| **Hole** | 1.09 × 10^20^ | 2.69 × 10^19^ | 7.30 × 10^19^ |

***Supplementary Note III***


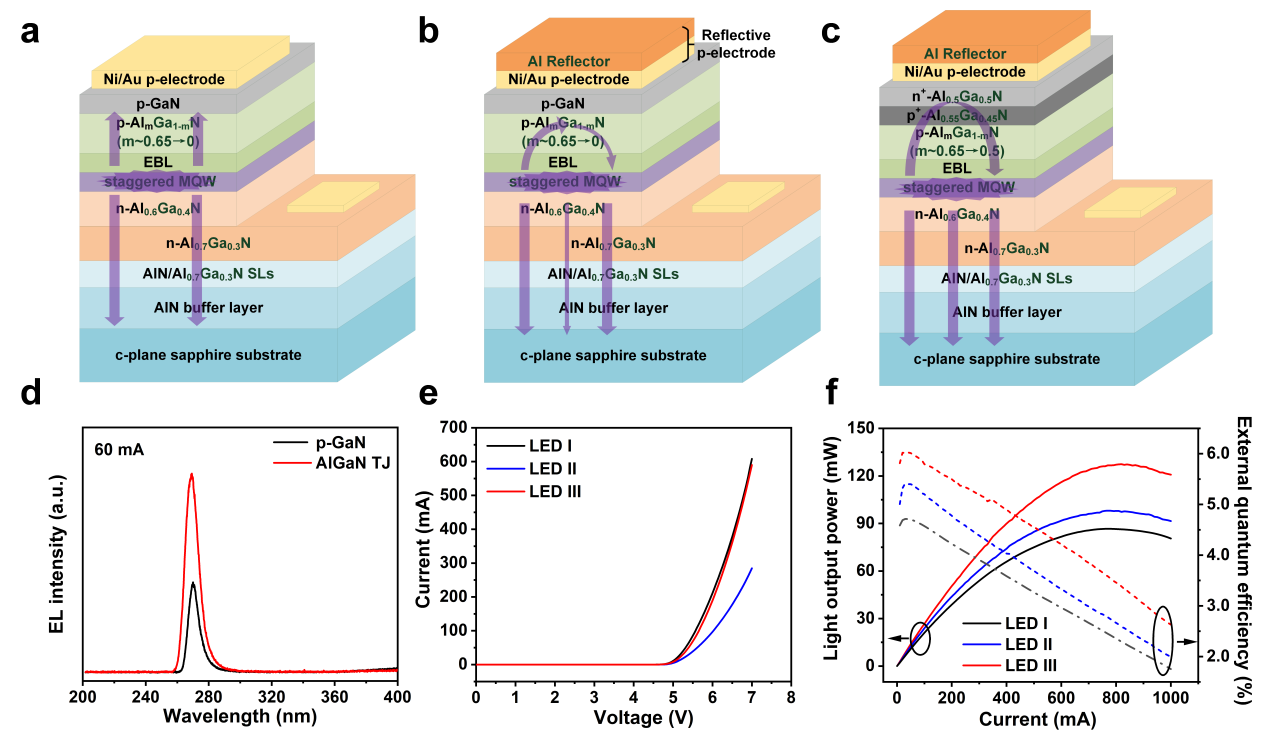


Fig. S12. Schematic illustration of (a) LED I, (b) LED II, and (c) LED III. (d) Normalized EL intensities of the epitaxial wafers with p-GaN and AlGaN TJ. (e) I-V characteristics, (f) experimental LOPs and EQEs of LED I, LED II and LED III under different injection currents. The sizes of LED I, LED II, and LED III are all 30 × 30 mil^2^.


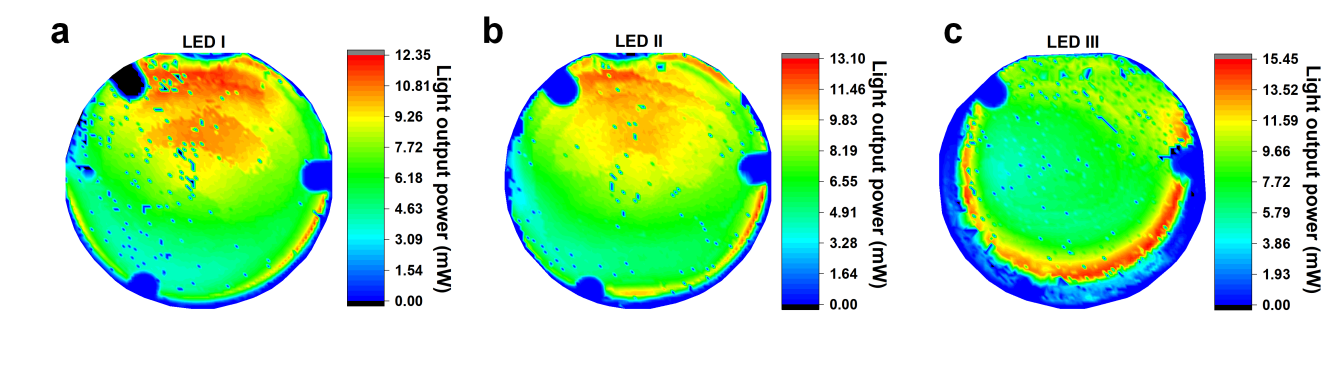


Fig. S13. Wafer-scaled LOPs of (a) LED I, (b) LED II and (c) LED III at 60 mA. The chips used to test the L-V-I characteristics are selected from the region in red color.

The schematic illustrations of LED I, LED II and LED III are shown in **Fig. S12(a-c)**. The AlGaN TJ consists of a 10 nm thick n^+^-Al_0.5_Ga_0.5_N layer and a 10 nm thick p^+^-Al_0.55_Ga_0.45_N layer. In addition, it is also demonstrated that the DUV wafer with AlGaN TJ possesses a higher EL intensity than that with p-GaN contact layer at an emission wavelength of ~270 nm in **Fig. S12(d)**. The current is plotted as a function of the operating voltage for LED-I, LED-II and LED-III in **Fig. S12(e)**. At 7 V, the current of LED-I, LED-II and LED-III is 610, 290 and 590 mA, respectively. The current of LED II is much lower than that of LED I, which can be attributed that the Al reflector on the p-electrode can deteriorate the current spreading of Ni/Au p-electrode^14, 15^. For LED III, the high-probability tunneling in the AlGaN TJ structure can effectively enhance the carrier injection efficiency^16, 17^, which can neutralize the negative impact accompanied by Al reflector. Therefore, the current of LED III is closed to that of LED I and both of them are higher than that of LED II at 7 V. LOP-I characteristic curves of LED I, LED II and LED III are exhibited in **Fig. S12(f)**. At 850 mA, the LOP of LED III is 127 mW, demonstrating 47.8% and 30.5% higher than that of LED I (85.9 mW) and LED II (97.3 mW). The peak EQE of LED I, II and III is 4.7, 5.4 and 6.0 %, respectively. Through comparative analysis, the enhancement of LOP realized by the AlGaN TJ is 2.6 times of that realized by the Al reflector on p-electrode. The locations of all chips on LED I, II, and III and corresponding LOPs are plotted in **Fig S13(a-c)**.

***Supplementary Note IV***


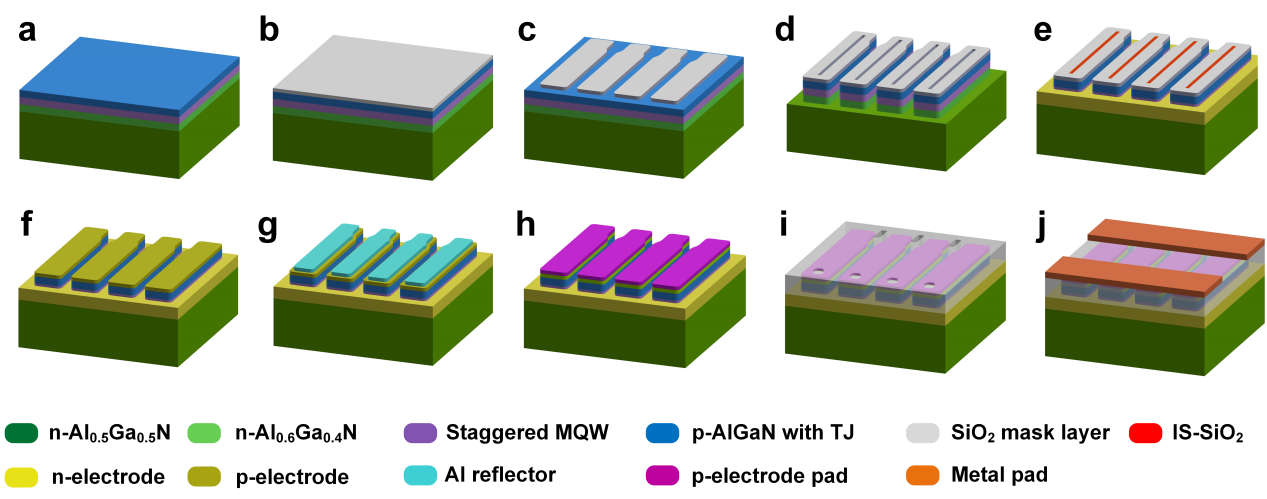


Fig. S14. Schematic illustration of the standard fabrication process for LED IV with cuboid IS-SiO_2_.

**Fig. S14(a-j)** illustrates the standard fabrication process of the LED IV with cuboid IS-SiO_2_. The wafer is purged with a mixed aqueous solution of HCl and H_2_O_2_ to remove the organic residue in **Fig. S14(a)**. Then, a 300-nm-thick SiO_2_ film is deposited on the cleaned wafer at 250 ℃ via using PECVD in **Fig. S14(b)**, followed by the combination of photolithography and wet etching processes using buffered oxide etch (BOE) solution (HF: NH_4_F = 6:1) for 50 s to achieve the patterned SiO_2_ mask layer in **Fig. S14(c)**. The mesa is etched by using the combination of photolithography and ICP etching with BCl_3_/Cl_2_ gas chemistry until n-Al_0.6_Ga_0.4_N ESL is exposed. The SiO_2_ mask layer is etched by using ICP based on SF_6_/Ar/N_2_ gas chemistry until the AlGaN TJ is exposed, followed by further ICP etching in the atmosphere of BCl_3_/Cl_2_/Ar to expose the c-plane sapphire in **Fig. S14(d)**. The etching duration and depth are 7800 s and ~6 μm, respectively. The etching structures are stuffed with SiO_2_ via using the PECVD. The deposition temperature, pressure, RF power and active gas flow ratio (N_2_O/10% SiH_4_) is kept at 300 ℃, 850 mTorr, 50 W and 33.3, respectively. Then, the Ti/Al/Ti/Au (30/200/30/1000 nm) multilayers are deposited on the exposed n-Al_0.6_Ga_0.4_N as n-electrode and thermally annealed in N_2_ at 960 ℃ for 120 s in **Fig. S14(e)**. After removing the SiO_2_ mask layer, the Ni/Au (30/50 nm) films for the p-electrode are deposited and thermally annealed in O_2_ at 650 ℃ for 70 s in **Fig. S14(f)**. Sequentially, the Al film and four-pair Ti/Ni metal stacks are deposited on the Ni/Au multilayers as p-electrode reflector and p-pad by using PECVD, respectively, as shown in **Fig. S14(g-h)**. Next, a 200-nm-thick SiO_2_ passivation layer is deposited on the mesa, followed by the ICP etching based on SF_6_/Ar/N_2_ gas chemistry to form n- and p-via holes in **Fig. S14(i)**. Finally, a 250-nm-thick metal layer of Au/Sn alloys are deposited on the n- and p-via holes as metal pads in **Fig. S14(j)**. The optical parameters of the metal materials used to fabricate the electrodes of DUV LEDs are shown in **Table S5**.

| Table S5. Optical parameters of the common metal materials used to fabriacte the electrodes of DUV LEDs^18-22^. | | | | |
| --- | --- | --- | --- | --- |
| **Materials** | **Refractive Index** | **[Extinction](https://baike.baidu.com/item/extinction/51002538?fromModule=lemma_inlink) [Coefficient](https://baike.baidu.com/item/coefficient/9517815?fromModule=lemma_inlink)** | **Reflectivity (%)** | **Work Function (eV)** |
| **Argentum (Ag)** | 1.3709 | 1.2596 | 23.920 | 4.26 |
| **Aluminium (Al)** | 0.17300 | 2.5760 | 91.363 | 4.28 |
| **Chromium (Cr)** | 1.4400 | 2.1800 | 46.198 | 4.50 |
| **Titanium (Ti)** | 1.1951 | 1.3462 | 27.904 | 4.33 |
| **Vanadium(V)** | 1.0249 | 2.5494 | 61.324 | 4.30 |
| **[Aurum](https://baike.baidu.com/item/Aurum/62174686?fromModule=lemma_inlink) (Au)** | 1.4119 | 1.7734 | 36.985 | 5.10 |
| **Nickel (Ni)** | 1.4103 | 2.9196 | 60.644 | 5.12 |
| **Platinum (Pt)** | 1.2107 | 2.5369 | 57.231 | 5.65 |


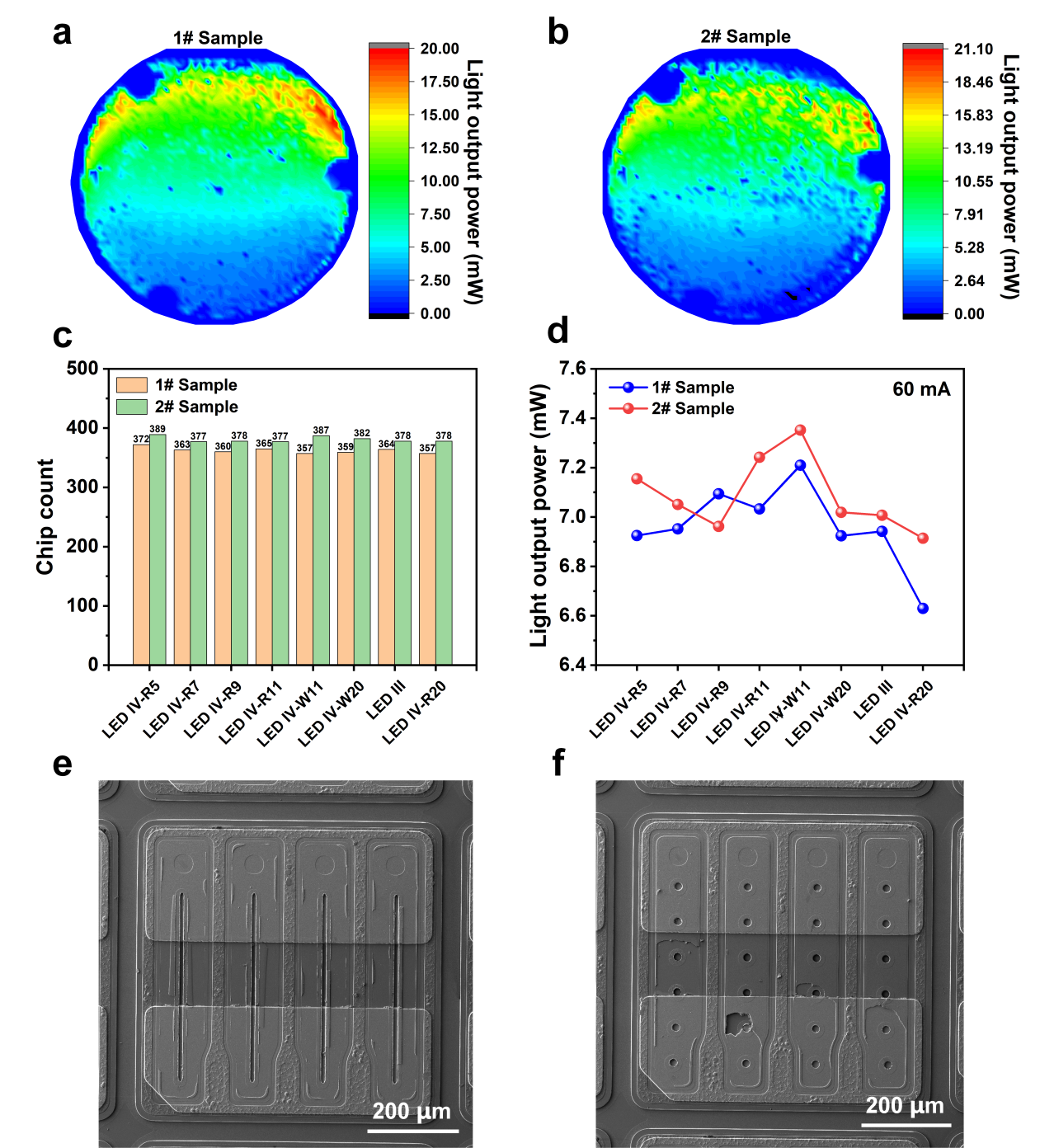


Fig. S15. (a-b) Wafer-scaled emission performance of the DUV LEDs with IS-SiO_2_. The chips used to test the L-V-I characteristics are selected from the regions in red color. (c) Chip count of LED III, LED RX and LED WY samples. (d) Average LOPs of LED III, LED RX and LED WY samples at 60 mA. Top-view SEM images of imperfect DUV LED with (e) cuboid and (f) columnar IS-SiO_2_.

LED III, LED RX and LED WY samples can be all fabricated on the same wafer owing to the mask layout. We use two DUV wafers with TJ, named as 1#Sample and 2#Sample, to implement the wafer-scaled emission performance measurement of DUV LEDs. The DUV wafers with TJ used in this work are all grown in the same batch. **Fig. S15(a-b)** illustrates the wafer-scaled emission performance of 1#Sample and 2#Sample, respectively. Obviously, the LOP distributions of 1#Sample and 2#Sample are both very poor. The numbers of tested chips for LED III, LED RX and LED WY samples are shown in **Fig. S15(c).** Furthermore, we calculated the average LOPs of LED III, LED RX and LED WY samples at 60 mA **[Fig. S15(d)]**, which are also exhibited in **Table S6**. The non-uniform distribution of LOPs makes their [arithmetic](https://cn.bing.com/dict/search?q=arithmetic&FORM=BDVSP6&cc=cn) [mean](https://cn.bing.com/dict/search?q=mean&FORM=BDVSP6&cc=cn) failed in [substantially](https://cn.bing.com/dict/search?q=substantially&FORM=BDVSP6&cc=cn) representing the true emission performances of the devices. However, the average LOP of LED IV-W11 samples is still the highest among all the tested DUV LEDs.

| Table S6. [Arithmetic](https://cn.bing.com/dict/search?q=arithmetic&FORM=BDVSP6&cc=cn) [mean](https://cn.bing.com/dict/search?q=mean&FORM=BDVSP6&cc=cn) of wafer-scaled LOP for LED III, LED RX and LED WY at 60 mA. | | | | | | | | |
| --- | --- | --- | --- | --- | --- | --- | --- | --- |
| **LOP (mW)** | **LED IV-R5** | **LED IV-R7** | **LED IV-R9** | **LED IV-R11** | **LED IV-W11** | **LED IV-W20** | **LED III** | **LED IV-R20** |
| **1#Sample** | 6.925 | 6.952 | 7.094 | 7.033 | 7.210 | 6.924 | 6.942 | 6.630 |
| **2#Sample** | 7.155 | 7.051 | 6.962 | 7.242 | 7.352 | 7.019 | 7.007 | 6.914 |

Top-view SEM image of imperfect DUV LED with IS-SiO_2_ can partly used to reveal the reasons why the LOP distributions of 1#Sample and 2#Sample are so poor. **Fig. S15(e-f)** shows the top-view SEM image of imperfect DUV LEDs with cuboid and columnar IS-SiO_2_, respectively. It is very easily found that there exists very obvious exfoliation of surface epitaxial layer in the DUV LED with cuboid IS-SiO_2_ **[Fig. S15(e)]**, even the part of active regions are peeled from the DUV LED, as shown in **Fig. S15(f)**. Compared to the LOP distributions of LED III, LED RX and LED WY, those of Structure A, B, and C are comparatively uniform in **Fig. S4**. It proves that the metal stacks used for p-electrode have enough strong [structural](https://cn.bing.com/dict/search?q=Structural&FORM=BDVSP6&cc=cn) [robustness](https://cn.bing.com/dict/search?q=Robustness&FORM=BDVSP6&cc=cn). However, the LOP distributions of the DUV LEDs with TJ are all very poor. A reasonable interpretation for this case is that the exfoliation of surface epitaxial layer in **Fig. S14(a-c)** and **Fig. S15(e-f)** may be ascribed to fragile TJ structure in LED III, LED RX, and LED WY. In addition, the harsh dry etching process used to fabricate the IS-SiO_2_ should be responsible for the loss of active region in **Fig. S15(f)**. The etching pits with larger size are formed via using multiple dry etching on the those with smaller size. For example, if we need a etching pit with a radius of 7 μm, we should first obtain a etching pit with a radius of 5 μm. Then, the inner sidewall of the obtained etching pit is further etched so that radius of the new etching pit is extended from 5 to 7 μm. Therefore, the size of etching pit is larger with increasing etching processes, thereby leading to unintentional damage to the active region of device. To further prove that the AlGaN TJ is vulnerable to the harsh etching condition during the fabrication of IS-SiO_2_, we prepared a DUV wafer with p-GaN contact layer with an emission wavelength of 275 nm , as shown in **Fig. S16(a)**. **Fig. S16(b)** shows the wafer-scaled emission performance of the DUV LEDs with p-GaN contact layer and IS-SiO_2_ at 100 mA. It is found that the DUV LEDs with p-GaN contact layer have the better yields of LOP than those with AlGaN TJ contact layer in **Fig. S15(a-b)**. Obviously, p-GaN is more robust than AlGaN TJ during the etching.


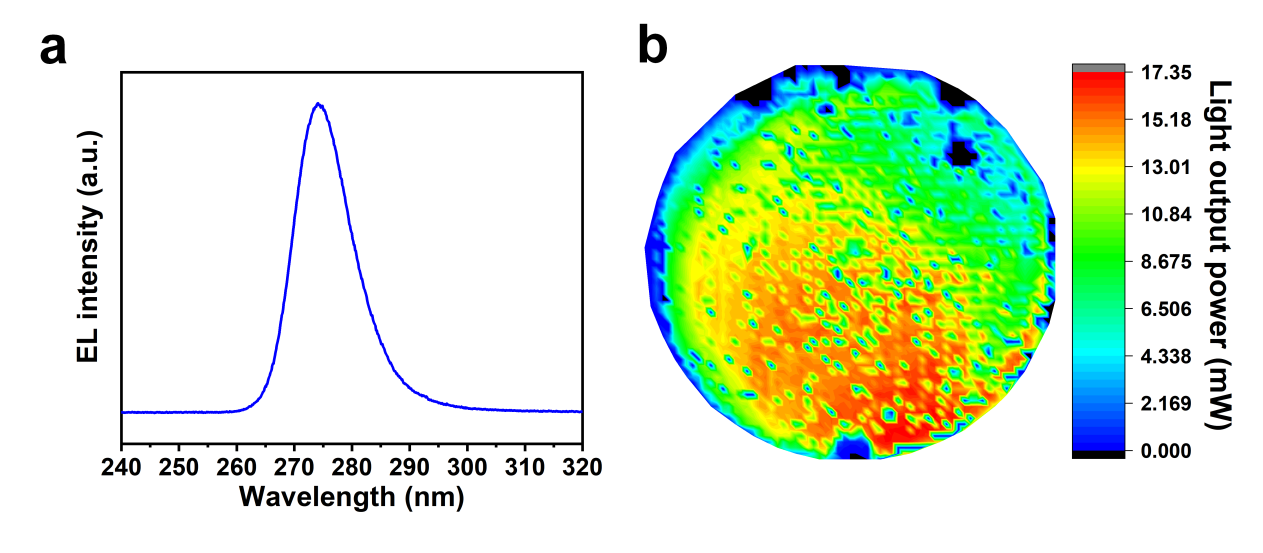


Fig. S16. (a) Normalized EL intensity of the DUV wafer with p-GaN contact layer at 100 mA. (b) Wafer-scaled emission performance of the DUV LEDs with p-GaN contact layer and IS-SiO_2_ at 100 mA. The chip size is 30 × 30 mil^2^.

**Supplementary references**

1. Wang M, Wu F, Yao Y, Zollner C, Iza M*, et al.* 10.6% external quantum efficiency germicidal UV LEDs grown on thin highly conductive n-AlGaN. *Applied Physics Letters* **123** (2023).

2. Matsukura Y, Inazu T, Pernot C, Shibata N, Kushimoto M*, et al.* Improving light output power of AlGaN-based deep-ultraviolet light-emitting diodes by optimizing the optical thickness of p-layers. *Applied Physics Express* **14**, 084004 (2021).

3. Pandey A, Shin WJ, Gim J, Hovden R, Mi Z. High-efficiency AlGaN/GaN/AlGaN tunnel junction ultraviolet light-emitting diodes. *Photonics Research* **8** (2020).

4. Yu H, Memon MH, Jia H, Ding Y, Xiao S*, et al.* Deep‐Ultraviolet LEDs Incorporated with SiO_2_‐Based Microcavities Toward High‐Speed Ultraviolet Light Communication. *Advanced Optical Materials* **10** (2022).

5. Zhao J, Li Q, Tan Q, Liang T, Zhou W*, et al.* Ring geometric effect on the performance of AlGaN-based deep-ultraviolet light-emitting diodes. *Optics Express* **32**, 1275-1285 (2024).

6. Wang L, Xu F, Lang J, Wang J, Zhang L*, et al.* Improving Light Extraction Efficiency of AlGaN-Based Deep Ultraviolet Light-Emitting Diodes by Combining Thinning p-AlGaN/p-GaN Layer With Ni/Au/Al High-Reflectivity Electrodes. *IEEE Photonics Journal* **15**, 1-5 (2023).

7. Wang L, Xu F, Lang J, Wang J, Zhang L*, et al.* Transparent p-type layer with highly reflective Rh/Al p-type electrodes for improving the performance of AlGaN-based deep-ultraviolet light-emitting diodes. *Japanese Journal of Applied Physics* **62**, 030904 (2023).

8. Sadaf SM, Zhao S, Wu Y, Ra YH, Liu X*, et al.* An AlGaN Core–Shell Tunnel Junction Nanowire Light-Emitting Diode Operating in the Ultraviolet-C Band. *Nano Letters* **17**, 1212-1218 (2017).

9. Zhang S, He R, Duo Y, Chen R, Wang L*, et al.* Plasmon-enhanced deep ultraviolet Micro-LED arrays for solar-blind communications. *Optics Letters* **48**, 3841-3844 (2023).

10. Xiao S, Yu H, Memon MH, Jia H, Luo Y*, et al.* In-Depth Investigation of Deep Ultraviolet MicroLED Geometry for Enhanced Performance. *IEEE Electron Device Letters* **44**, 1520-1523 (2023).

11. Li D, Liu SF, Qian ZY, Liu QF, Zhou K*, et al.* Deep-Ultraviolet Micro-LEDs Exhibiting High Output Power and High Modulation Bandwidth Simultaneously. *Advanced Materials* **34** (2022).

12. Wang S, Long H, Zhang Y, Chen Q, Dai J*, et al.* Monolithic integration of deep ultraviolet LED with a multiplicative photoelectric converter. *Nano Energy* **66** (2019).

13. Lughi V, Clarke DR. Defect and stress characterization of AlN films by Raman spectroscopy. *Applied Physics Letters* **89** (2006).

14. Maeda N, Jo M, Hirayama H. Improving the Efficiency of AlGaN Deep‐UV LEDs by Using Highly Reflective Ni/Al p‐Type Electrodes. *physica status solidi (a)* **215** (2018).

15. Lee TH, Park TH, Shin HW, Maeda N, Jo M*, et al.* Smart Wide‐Bandgap Omnidirectional Reflector as an Effective Hole‐Injection Electrode for Deep‐UV Light‐Emitting Diodes. *Advanced Optical Materials* **8** (2019).

16. Kuhn C, Sulmoni L, Guttmann M, Glaab J, Susilo N*, et al.* MOVPE-grown AlGaN-based tunnel heterojunctions enabling fully transparent UVC LEDs. *Photon Res* **7** (2019).

17. Xiao S, Yu H, Jia H, Memon MH, Wang R*, et al.* Performance evaluation of tunnel junction-based N-polar AlGaN deep-ultraviolet light-emitting diodes. *Opt Lett* **47** (2022).

18. Cheng F, Su P-H, Choi J, Gwo S, Li X*, et al.* Epitaxial Growth of Atomically Smooth Aluminum on Silicon and Its Intrinsic Optical Properties. *ACS Nano* **10**, 9852-9860 (2016).

19. Palm KJ, Murray JB, Narayan TC, Munday JN. Dynamic Optical Properties of Metal Hydrides. *ACS Photonics* **5**, 4677-4686 (2018).

20. Babar S, Weaver JH. Optical constants of Cu, Ag, and Au revisited. *Applied Optics* **54** (2015).

21. Werner WSM, Glantschnig K, Ambrosch-Draxl C. Optical Constants and Inelastic Electron-Scattering Data for 17 Elemental Metals. *Journal of Physical and Chemical Reference Data* **38**, 1013-1092 (2009).

22. Johnson PB, Christy RW. Optical constants of transition metals: Ti, V, Cr, Mn, Fe, Co, Ni, and Pd. *Physical Review B* **9**, 5056-5070 (1974).
